# FSP1-positive fibroblasts are adipogenic niche and regulate adipose homeostasis

**DOI:** 10.1371/journal.pbio.2001493

**Published:** 2018-08-06

**Authors:** Rui Zhang, Yuan Gao, Xiaotong Zhao, Mei Gao, Yanjun Wu, Yingying Han, Yuemei Qiao, Zheng Luo, Li Yang, Jianfeng Chen, Gaoxiang Ge

**Affiliations:** 1 State Key Laboratory of Cell Biology, CAS Center for Excellence in Molecular Cell Science, Shanghai Institute of Biochemistry and Cell Biology, Chinese Academy of Sciences, Shanghai, China; 2 CAS Key Laboratory of Systems Biology, CAS Center for Excellence in Molecular Cell Science, Shanghai Institute of Biochemistry and Cell Biology, Chinese Academy of Sciences, Shanghai, China; 3 Innovation Center for Cell Signaling Network, CAS Center for Excellence in Molecular Cell Science, Shanghai Institute of Biochemistry and Cell Biology, Chinese Academy of Sciences, Shanghai, China; 4 University of Chinese Academy of Sciences, Beijing, China; 5 CAS Key Laboratory of Computational Biology, CAS-MPG Partner Institute for Computational Biology, CAS Center for Excellence in Brain Science and Intelligence Technology, Shanghai Institutes for Biological Sciences, Chinese Academy of Sciences, Shanghai, China; University of California at Los Angeles, United States of America

## Abstract

Adipocyte progenitors reside in the stromal vascular fraction (SVF) of adipose tissues that are composed of fibroblasts, immune cells, and endothelial cells. It remains to be elucidated how the SVF regulates adipocyte progenitor fate determination and adipose homeostasis. Here, we report that fibroblast-specific protein-1 (FSP1)^+^ fibroblasts in the SVF are essential to adipose homeostasis. FSP1^+^ fibroblasts, devoid of adipogenic potential, are adjacent to the preadipocytes in the SVF. Ablation of FSP1^+^ fibroblasts in mice severely diminishes fat content of adipose depots. Activation of canonical Wnt signaling in the FSP1^+^ fibroblasts results in gradual loss of adipose tissues and resistance to diet-induced obesity. Alterations in the FSP1^+^ fibroblasts reduce platelet-derived growth factor (PDGF)-BB signaling and result in the loss of preadipocytes. Reduced PDGF-BB signaling, meanwhile, impairs the adipogenic differentiation capability of preadipocytes by regulating matrix metalloproteinase (MMP) expression, extracellular matrix remodeling, and the activation of Yes-associated protein (YAP) signaling. Thus, FSP1^+^ fibroblasts are an important niche essential to the maintenance of the preadipocyte pool and its adipogenic potential in adipose homeostasis.

## Author summary

White adipose tissue (WAT), which consists mostly of adipocytes, is not only a passive energy storage but also an active metabolic and endocrine organ in the body. The importance of maintaining proper adipose mass is emphasized by the fact that both adipose tissue excess—in obese individuals—and deficiency have adverse metabolic consequences. In order to maintain the number of adipocytes, there is a continuous turnover from preadipocytes in adults. Like any other adult stem cells and progenitor cells, cell fate and differentiation capability of preadipocytes are tightly regulated by a highly specialized niche. However, what constitutes the preadipocyte niche, and how the niche regulates preadipocyte function and adipose homeostasis, remain poorly known. In this study, we have identified fibroblast-specific protein-1 (FSP1)^+^ fibroblasts in the WAT stromal vascular fraction (SVF) of mice as the niche for preadipocytes. We show that FSP1^+^ fibroblasts with aberrant Wnt signaling fail to maintain the preadipocyte pool and its differentiation potential, resulting in loss of adipose tissue. We conclude that FSP1^+^ fibroblasts are a niche for preadipocytes and regulate adipose tissue homeostasis in adult mice.

## Introduction

Adult adipose tissue contains adipocyte progenitors that are critical to adipose homeostatic turnover as well as adaptive hyperplastic expansion and regeneration [1–4]. Peroxisome proliferator-activated receptor-γ (PPARγ)^+^ adipocyte progenitors and preadipocytes—characteristic of cell surface markers, e.g., cluster of differentiation 34 (CD34) and stem cell antigen-1 (Sca1)—reside in the adipose vasculature expressing mural cell markers α-smooth muscle actin (αSMA), platelet-derived growth factor receptor-β (PDGFR-β), and neural glial antigen 2 (NG2) [1,2,5,6]. Stromal vascular fraction (SVF)-resident preadipocytes are highly committed and are capable of proliferating and differentiating into mature adipocytes in vitro and in vivo [1–3]. Cell fate and differentiation capability of the preadipocytes are regulated by signaling pathways and transcriptional and epigenetic programs. Wnt signaling plays crucial roles during development [7]. Activation of β-catenin-dependent canonical Wnt signaling in the preadipocytes inhibits adipogenesis [5,8,9].

Adipogenic potential of preadipocytes is determined not only by their intrinsic properties but also by the surrounding microenvironment. Diet-induced obesity is regulated by the adipose microenvironment, but not by cell-intrinsic mechanisms, highlighting the importance of microenvironmental regulation in adipose homeostasis [10]. Adipose tissues contain multiple types of stromal cells, including fibroblasts, endothelial cells, and immune cells. While macrophage-related chronic inflammation in the adipose tissues was reported to play a crucial role in the development of obesity and obesity-related insulin resistance [11,12], roles of other cell types in preadipocyte fate determination and adipose homeostasis are largely unknown. Fibroblasts are resident cell types in the adipose tissues with a mesenchymal lineage origin. Fibroblast-specific protein 1 (FSP1; also known as S100A4) is a reliable marker for detecting tissue-resident fibroblasts, in addition to other mesenchymal markers αSMA, PDGFR-β, and NG2 [13–15]. While the roles of fibroblasts in adipose homeostasis remain elusive, fibroblasts have multifaceted regulatory roles in tissue morphogenesis, wound healing, fibrosis, and cancer by modulating the behavior and functions of epithelial cells and stem cells [13,15–17].

Here, we report that FSP1^+^ fibroblasts in the SVF are a niche for adipogenesis. Activation of canonical Wnt signaling in the FSP1^+^ fibroblasts or ablation of FSP1^+^ fibroblasts disturbs adipose homeostasis and results in loss of adiposity. Alterations in the FSP1^+^ fibroblasts resulted in decreased platelet-derived growth factor (PDGF) expression. On the one hand, PDGF maintains the preadipocyte number. On the other hand, PDGF regulates the adipogenic potential of preadipocytes by regulating extracellular matrix remodeling in the microenvironment and Yes-associated protein (YAP) activation. Thus, FSP1^+^ fibroblasts are a niche for preadipocytes orchestrating the functions of preadipocytes and their microenvironment.

## Results

### FSP1^+^ fibroblasts are adjacent to preadipocytes without adipogenic potential

**Fig 1:**
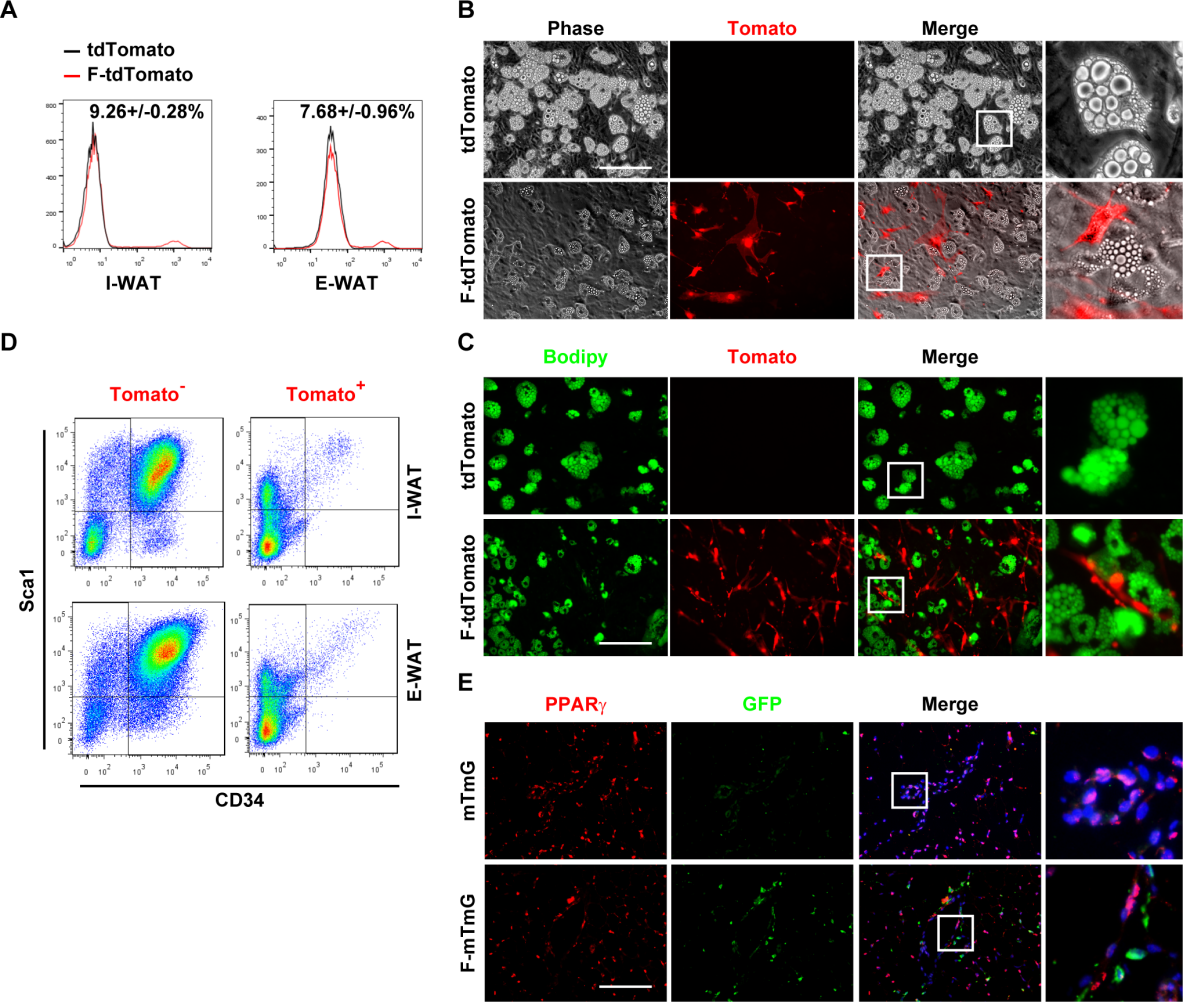
FSP1^+^ fibroblasts localize adjacent to preadipocytes without adipogenic potential. (A) FACS analyses of tdTomato^+^ cells in SVF cells isolated from I-WAT and E-WAT of 4-month-old *Rosa26-tdTomato* (mT) and *Fsp1*-*Cre*; *tdTomato* (F-mT) mice. (B, C) SVF cells isolated from *Rosa26-tdTomato* (mT) and *Fsp1*-*Cre*; *tdTomato* (F-mT) I-WAT were adipogenically induced. Cells were stained with Bodipy 493/503 (panel C). Scale bar: 200 μm. (D) FACS analyses of CD34^+^Sca1^+^ cells in I-WAT and E-WAT SVF of 4-month-old *Fsp1*-*Cre*; *tdTomato* mice. (E) Immunofluorescent staining of GFP and PPARγ on I-WAT sections of 4-month-old mTmG and *Fsp1*-*Cre*; *mTmG* (F-mTmG) mice. Scale bar: 200 μm. CD34, cluster of differentiation 34; E-WAT, epididymal white adipose tissue; FACS, fluorescence-activated cell sorting; FSP1, fibroblast-specific protein-1; GFP, green fluorescent protein; I-WAT, inguinal white adipose tissue; PPARγ, peroxisome proliferator-activated receptor-γ; Sca1, stem cell antigen 1; SVF, stromal vascular fraction.

Preadipocytes express mesenchymal markers αSMA, PDGFR-β, and NG2. To investigate whether FSP1^+^ fibroblasts are part of the adipocyte lineage, *Fsp1*-*Cre* mice [15] were crossed with *Rosa26*-floxed stop *tdTomato* reporter mice. Only very weak tdTomato signal was detected in the inguinal and epididymal white adipose tissues (I-WATs and E-WATs) from the *Fsp1*-*Cre*;*tdTomato* compound mice on a normal-chow diet (ND) (S1A Fig), in contrast to strong tdTomato signal in the tail tip fibroblasts (S1A and S1B Fig). Fluorescence-activated cell sorting (FACS) analysis indicated that a small percentage of the SVF cells, which expressed fibroblast markers αSMA and vimentin (S1C and S1D Fig), expressed tdTomato (Fig 1A). Upon high-fat diet (HFD)-induced obesity, percentage of the FSP1^+^/tdTomato^+^ SVF cells increased (I-WAT: 9.26% ± 0.28% [ND] versus 18.17% ± 1.36% [HFD]; E-WAT: 7.68% ± 0.96% [ND] versus 12.33 ± 0.67% [HFD]). SVF preadipocytes differentiated to mature adipocytes upon adipogenic induction (Fig 1B and 1C). tdTomato^+^ SVF cells, however, did not differentiate to adipocytes (Fig 1B and 1C). Consistently, CD34^+^Sca1^+^ preadipocytes were enriched in the tdTomato^−^, but not the tdTomato^+^, SVF populations (Fig 1D and S1E Fig). To gain a better idea of the spatial localization of the FSP1^+^ cells, immunostaining of PPARγ (preadipocytes) and green fluorescent protein (GFP; FSP1^+^ fibroblasts) was performed on the white adipose tissue (WAT) sections of the *Fsp1*-*Cre*;*Rosa26*-flox-membrane-targeted Tomato-flox-membrane-targeted GFP (F-*mTmG*) mice. PPARγ^+^ and GFP^+^ SVF cells were adjacent to each other but mutually exclusive (Fig 1E). These data collectively suggested that the FSP1^+^ fibroblasts were not in the adipocyte lineage but were localized in the microenvironment of preadipocytes in the SVF.

### Activation of canonical Wnt signaling in FSP1^+^ fibroblasts results in loss of adiposity

**Fig 2:**
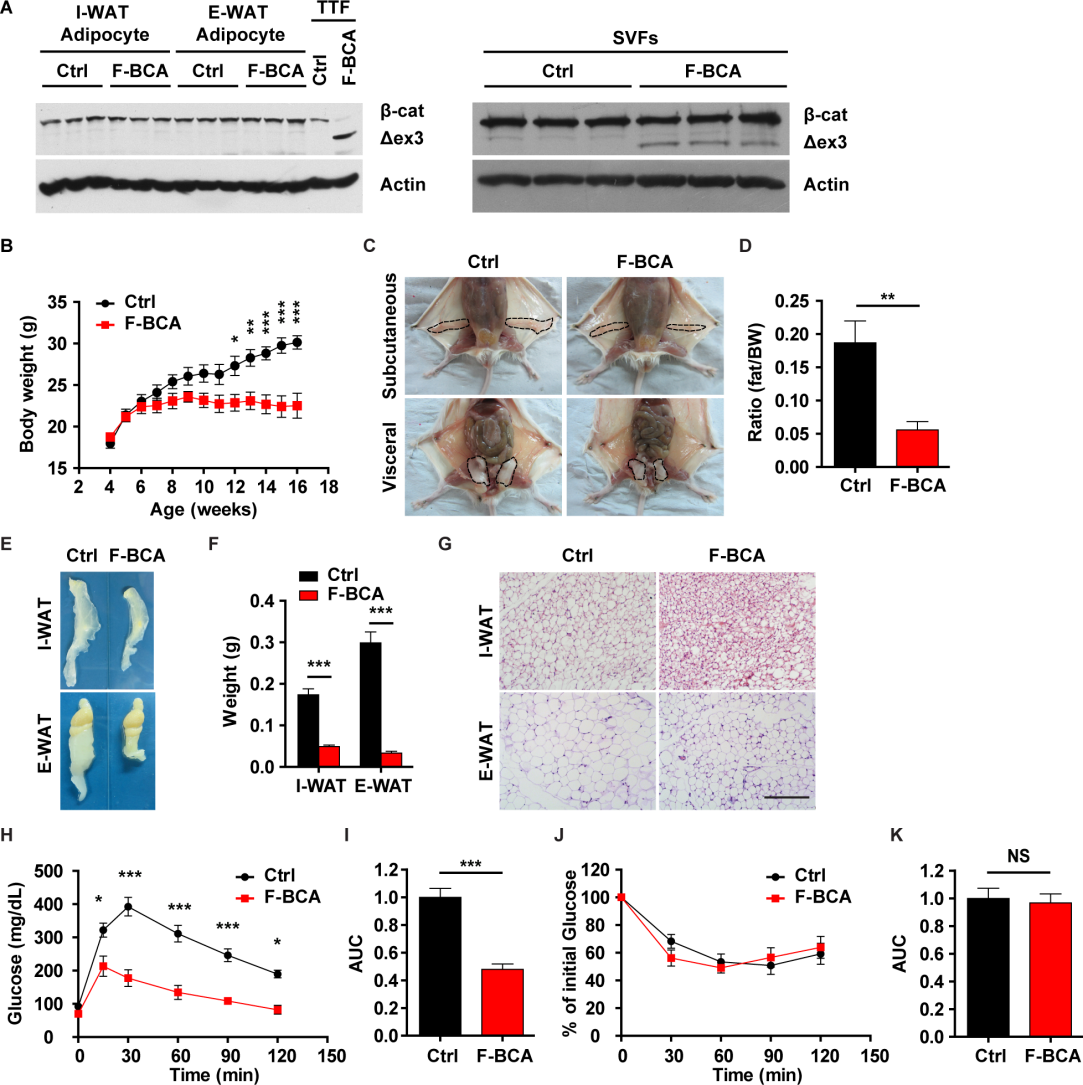
Activation of canonical Wnt signaling in FSP1^+^ fibroblasts disturbs adipose homeostasis. (A) Western blot analyses of β-catenin expression in adipocytes (left panels) and SVF cells (right panels) isolated from WAT from F-BCA compound mice and their littermates with WT, *Fsp1*-*Cre*, or *Ctnnb1*^exon 3 fl/+^ genotypes (Ctrl). TTFs were used as a positive Ctrl for activated form β-catenin expression. (B) Body weight of male F-BCA mice and their littermates on ND. *n* = 9 for male Ctrl mice, and *n* = 10 for male F-BCA mice. (C) Ventral view of subcutaneous and visceral adipose depots of male Ctrl and F-BCA littermates on ND at 4 months of age. Adipose depots are circled with dashed lines. (D) Percent body fat (NMR spectroscopy) of Ctrl and F-BCA littermates on ND at 4 months of age. *n* = 6 for male Ctrl and F-BCA mice. (E, F) Representative pictures and weight of the adipose tissues of F-BCA mice and their littermates on ND at 4 months of age. *n* = 8 for male Ctrl mice, and *n* = 14 for male F-BCA mice. (G) HE staining of WAT sections of 4-month-old F-BCA mice and their littermates on ND. Scale bar: 200 μm. (H) GTT of F-BCA mice and their littermates on ND at 4 months of age. *n* = 13 for male Ctrl mice, and *n* = 5 for male F-BCA mice. (I) Quantification of the AUC of the GTT relative to Ctrl group. (J) ITT of F-BCA mice and their littermates on ND at 4 months of age. *n* = 10 for male Ctrl mice, and *n* = 5 for male F-BCA mice. (K) Quantification of the AUC of the ITT relative to Ctrl group. Data are presented as mean ± SEM. Statistical analyses were performed with two-tailed unpaired student *t* test (panel D, F, I, and K) or two-way ANOVA followed by Bonferroni's multiple comparison test (panel B, H, and J). *p < 0.05; **p < 0.01; ***p < 0.001. Underlying data can be found in S1 Data. AUC, area under the curve; Ctrl, control; F-BCA, *Fsp1*-*Cre*;*Ctnnb1*^exon 3 fl/+^; FSP1, fibroblast-specific protein-1; GTT, glucose tolerance test; HE, hematoxylin–eosin; ITT, insulin tolerance test; ND, normal-chow diet; NMR, nuclear magnetic resonance; NS, not significant; SVF, stromal vascular fraction; TTF, tail tip fibroblast; WAT, white adipose tissue; WT, wild-type.

Wnt regulates adipose homeostasis by activating β-catenin in preadipocytes to inhibit their differentiation [8,9]. In addition to its direct inhibitory effect on preadipocyte differentiation [8], Wnt may regulate adipose homeostasis through cell types in the microenvironment, e.g., fibroblasts. Expression of β-catenin target genes were altered in the FSP1^+^/tdTomato^+^ fibroblasts upon HFD-induced obesity (S2A Fig). To investigate whether activation of Wnt signaling in FSP1^+^ fibroblasts would affect adipose homeostasis, *Ctnnb1*^exon 3 fl/+^ mice were crossed with *Fsp1*-*Cre* mice to generate the *Fsp1*-*Cre*;*Ctnnb1*^exon 3 fl/+^ (F-BCA) compound mice. *Fsp1*-*Cre*-driven deletion of exon 3 in β-catenin produced a smaller–molecular-weight β-catenin protein in tail tip fibroblasts (Fig 2A and S2B Fig). Despite the fact that the activated form of β-catenin was present in the SVF (Fig 2A and S2B–S2D Fig), there was no detectable activated form of β-catenin in the WAT or adipocytes from the F-BCA mice (Fig 2A and S2B Fig). These data further suggested that FSP1^+^ fibroblasts were not directly involved in the adipocyte lineage (Fig 1). In line with this notion, tdTomato^+^/FSP1^+^ SVF cells were negative for preadipocyte markers in F-BCA mice (S2E Fig).

*Fsp1*-*Cre*-driven activation of canonical Wnt signaling in fibroblasts did not affect embryonic development. F-BCA mice were born at expected Mendelian ratio. Mice with an *Fsp1*-*Cre* or *Ctnnb1*^exon 3 fl/+^ genotype were phenotypically similar to the wild-type (WT) littermates throughout the lifespan. Mice with a WT, *Fsp1*-*Cre*, or *Ctnnb1*^exon 3 fl/+^ genotype were therefore classified as controls in comparison to their F-BCA littermates. At weaning, F-BCA male mice were phenotypically normal, with similar amounts of adipose depots to the control mice (S3A–S3D Fig). Dissected I-WATs and E-WATs were similar in size to those from the control mice (S3C Fig). However, F-BCA male mice gain less weight after puberty compared with the control mice (Fig 2B). At 4 months of age, F-BCA male mice had less fat compared with the control mice (Fig 2C–2F). Dissected I-WATs and E-WATs were significantly smaller in size and weighed less in the F-BCA mice (Fig 2E and 2F). Histology inspection on adipose tissue sections showed that the F-BCA adipocyte diameter was significantly smaller (Fig 2G). F-BCA male mice at 8 months of age were virtually free of subcutaneous or visceral adipose depots (S3E–S3H Fig). The adipose phenotype was further confirmed by dissecting the WATs from the control and F-BCA male mice (S3G Fig) and by histology inspection on the adipose tissue sections (S3H Fig). Metabolic cage analyses revealed that the F-BCA male mice had similar food uptake, respiratory exchange ratio, and physical activity, but slightly reduced energy expenditure compared with the control mice (S4A–S4D Fig). Such defects in adipose homeostasis were also observed in the female F-BCA mice (S5 Fig).

WAT is an important metabolic regulator. Adipose defects observed in the F-BCA mice mimic lipodystrophy, a disorder accompanied by metabolic disturbances, including hyperglycemia, insulin resistance, and ectopic lipid deposition in secondary organs [8]. F-BCA male mice did not show the metabolic abnormality observed in the classical lipodystrophy mouse models. Rather activation of β-catenin in the FSP1^+^ fibroblasts offered metabolic benefits in the mice (Fig 2H–2K). F-BCA male mice were able to more efficiently clear glucose (Fig 2H and 2I) but retained the same insulin responsiveness as the control mice (Fig 2J and 2K). Livers weighed less, and no ectopic lipid deposition was observed in the livers in the F-BCA male mice (S4I–S4K Fig).

### F-BCA mice are resistant to diet-induced obesity

**Fig 3:**
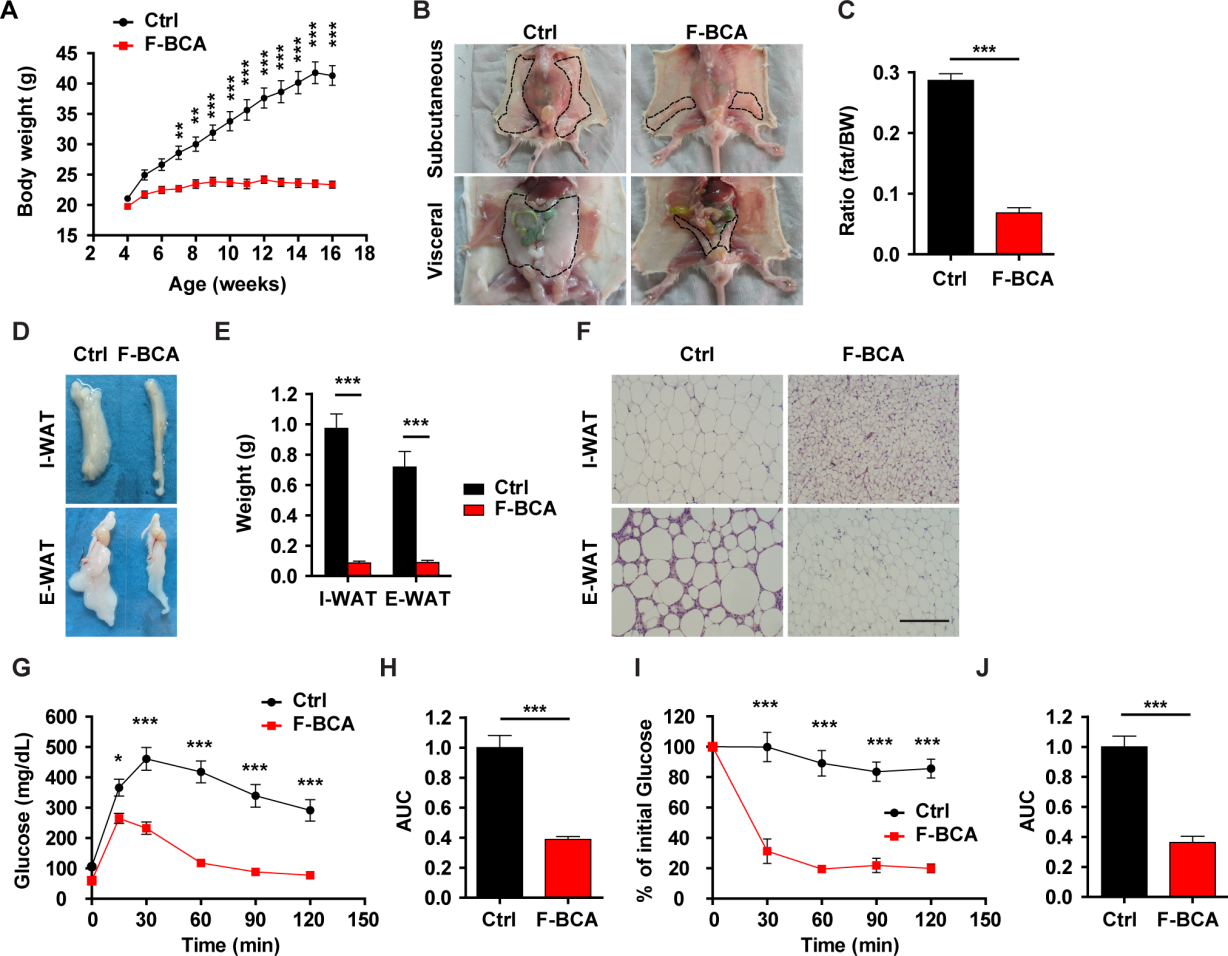
Activation of canonical Wnt signaling in FSP1^+^ fibroblasts protects mice from diet-induced obesity. (A) Body weight of male F-BCA mice and their control littermates on an HFD. *n* = 8 for male control mice, and *n* = 7 for male F-BCA mice. (B) Ventral view of subcutaneous and visceral adipose depots of control and F-BCA littermates on an HFD at 4 months of age. Adipose depots are circled with dashed lines. (C) Percent body fat (NMR spectroscopy) of control and F-BCA littermates on an HFD at 4 months of age. *n* = 8 for male control mice, and *n* = 7 for male F-BCA mice. (D, E) Representative pictures and weight of the adipose tissues of F-BCA mice and their control littermates on an HFD at the age of 4 months. *n* = 12 for male control mice, and *n* = 12 for male F-BCA mice. (F) HE staining of WAT sections of 4-month-old F-BCA mice and their control littermates. Scale bar: 200 μm. (G) GTT of F-BCA mice and their control littermates on an HFD at 4 months of age. *n* = 8 for male control mice, and *n* = 7 for male F-BCA mice. (H) Quantification of the AUC of the GTT relative to the control group. (I) ITT of F-BCA mice and their control littermates on an HFD at 4 months of age. *n* = 8 for male control mice, and *n* = 7 for male F-BCA mice. (J) Quantification of the AUC of the ITT relative to the control group. Data are presented as mean ± SEM. Statistical analyses were performed with two-tailed unpaired student *t* test (panel C, E, H, and J) or two-way ANOVA followed by Bonferroni's multiple comparison test (panel A, G, and I). *p < 0.05; **p < 0.01; ***p < 0.001. Underlying data can be found in S1 Data. AUC, area under the curve; Ctrl, control; E-WAT, epididymal white adipose tissue; F-BCA, *Fsp1*-*Cre*;*Ctnnb1*^exon 3 fl/+^; FSP1, fibroblast-specific protein-1; GTT, glucose tolerance test; HE, hematoxylin–eosin; HFD, high-fat diet; ITT, insulin tolerance test; I-WAT, inguinal white adipose tissue; NMR, nuclear magnetic resonance; NS, not significant; WAT, white adipose tissue.

Activation of preadipocytes drives adipocyte hyperplasia in diet-induced obesity [10]. Such a process is regulated by the adipose microenvironment but not by cell-intrinsic mechanisms [10]. To investigate whether activation of canonical Wnt signaling in the FSP1^+^ fibroblasts would affect diet-induced obesity, F-BCA male mice were fed with an HFD for 12 weeks. On HFD, F-BCA male mice consumed more food and had significantly enhanced respiratory exchange ratio and physical activity compared with the control mice (S4E–S4H Fig). Unlike the control mice, which accumulated a significant amount of fat upon HFD feeding (Fig 3A–3F), F-BCA male mice were resistant to HFD-induced obesity (Fig 3A–3F). Control mice fed with an HFD were glucose intolerant and insulin resistant (Fig 3G–3J). F-BCA mice on an HFD cleared glucose as efficiently as those on an ND (Fig 3G and 3H). F-BCA mice on an HFD were more sensitive to insulin (Fig 3I and 3J). Although control mice accumulated a large amount of fat in the liver upon HFD feeding, F-BCA mice were protected from HFD-induced steatosis (S4J and S4K Fig).

### FSP1^+^ fibroblasts maintain preadipocyte pool via PDGF signaling

**Fig 4:**
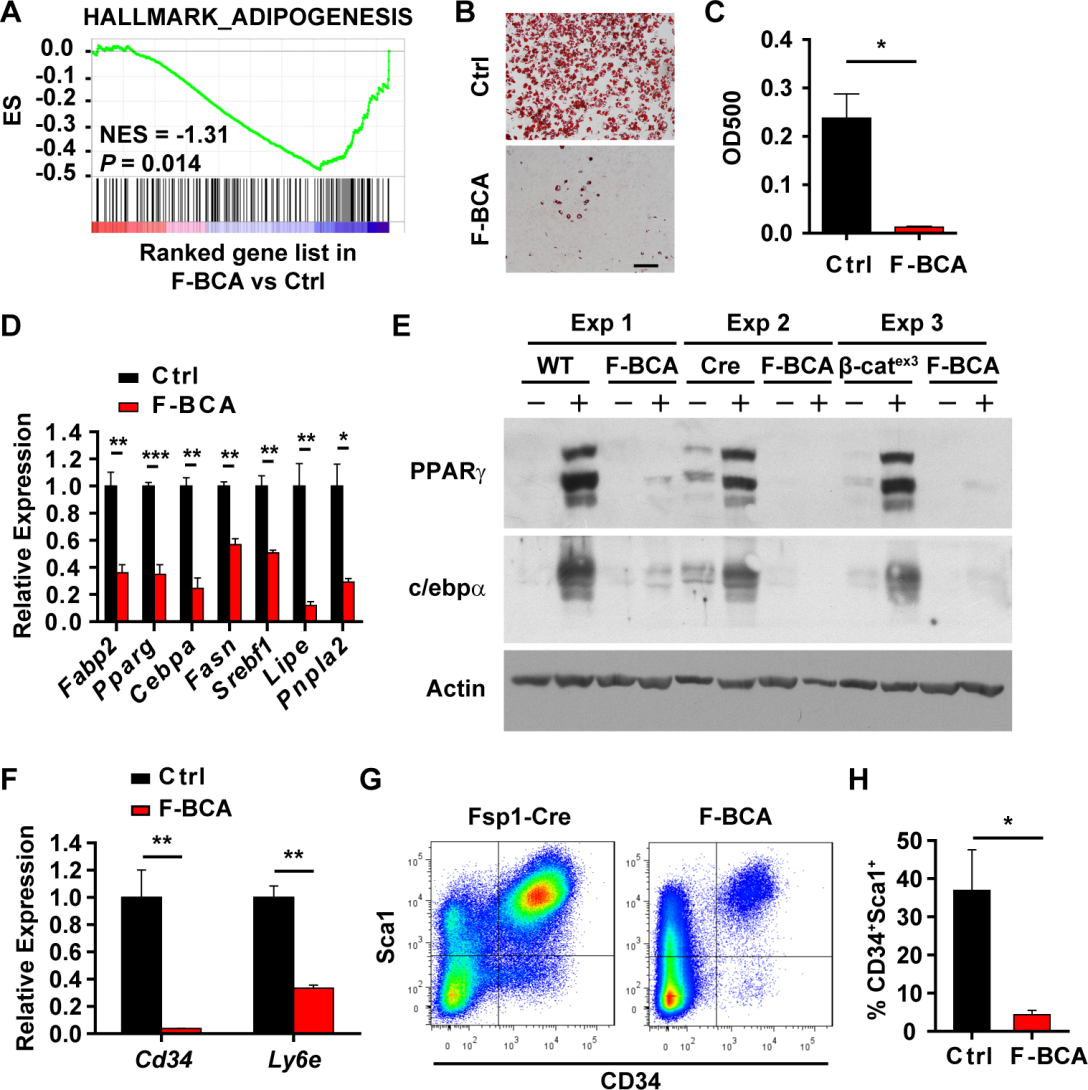
Activation of canonical Wnt signaling in FSP1^+^ fibroblasts reduces the number of preadipocytes and impairs adipogenesis. (A) GSEA data showing negative enrichment of adipogenesis signature in F-BCA SVF cells compared with the Ctrl SVF cells. (B, C) Ctrl and F-BCA SVF cells were subjected to adipogenic induction. Adipogenesis was assayed with Oil Red O staining (panel B). Scale bar: 100 μm. Oil Red O staining was quantitated by isopropanol extraction (*n* = 3) (panel C). (D) RT-PCR analyses of adipogenic marker expression in Ctrl and F-BCA SVF cells (*n* = 3). (E) Western blot analyses of PPARγ and c/ebpα in Ctrl and F-BCA SVF cells 6 days after adipogenic induction. (F) RT-PCR analyses of preadipocyte marker expression in Ctrl and F-BCA SVF cells (*n* = 3). (G, H) FACS analyses of CD34^+^Sca1^+^ cells in the SVF of 4-month-old Ctrl and F-BCA mice (*n* = 5). Data are presented as mean ± SEM. Statistical analyses were performed with two-tailed unpaired student *t* test. *p < 0.05; **p < 0.01; ***p < 0.001. Underlying data can be found in S1 Data. CD34, cluster of differentiation 34; Ctrl, control; F-BCA, *Fsp1*-*Cre*;*Ctnnb1*^exon 3 fl/+^; FACS, fluorescence-activated cell sorting; FSP1, fibroblast-specific protein-1; GSEA, gene set enrichment analysis; NES, normalized enrichment score; PPARγ, peroxisome proliferator-activated receptor-γ; RT-PCR, reverse transcription PCR; Sca1, stem cell antigen 1; SVF, stromal vascular fraction.

Activation of canonical Wnt signaling in the FSP1^+^ fibroblasts disturbed adipose homeostasis. Gene set enrichment analysis (GSEA) on gene expression of SVF cells indicated impaired adipogenic potential of the SVF cells isolated from the F-BCA mice (Fig 4A). Indeed, F-BCA SVF cells less efficiently differentiated into adipocytes upon adipogenic induction compared with the control SVF cells (Fig 4B–4E). Inefficient differentiation of F-BCA SVF cells may result from a reduced number of preadipocytes or from impaired differentiation potential of the preadipocytes. F-BCA SVF cells had lower expression levels of preadipocyte markers than the control SVF cells (Fig 4F). FACS analyses revealed reduced numbers of CD34^+^Sca1^+^ preadipocytes in the F-BCA SVF (Fig 4G and 4H).

**Fig 5:**
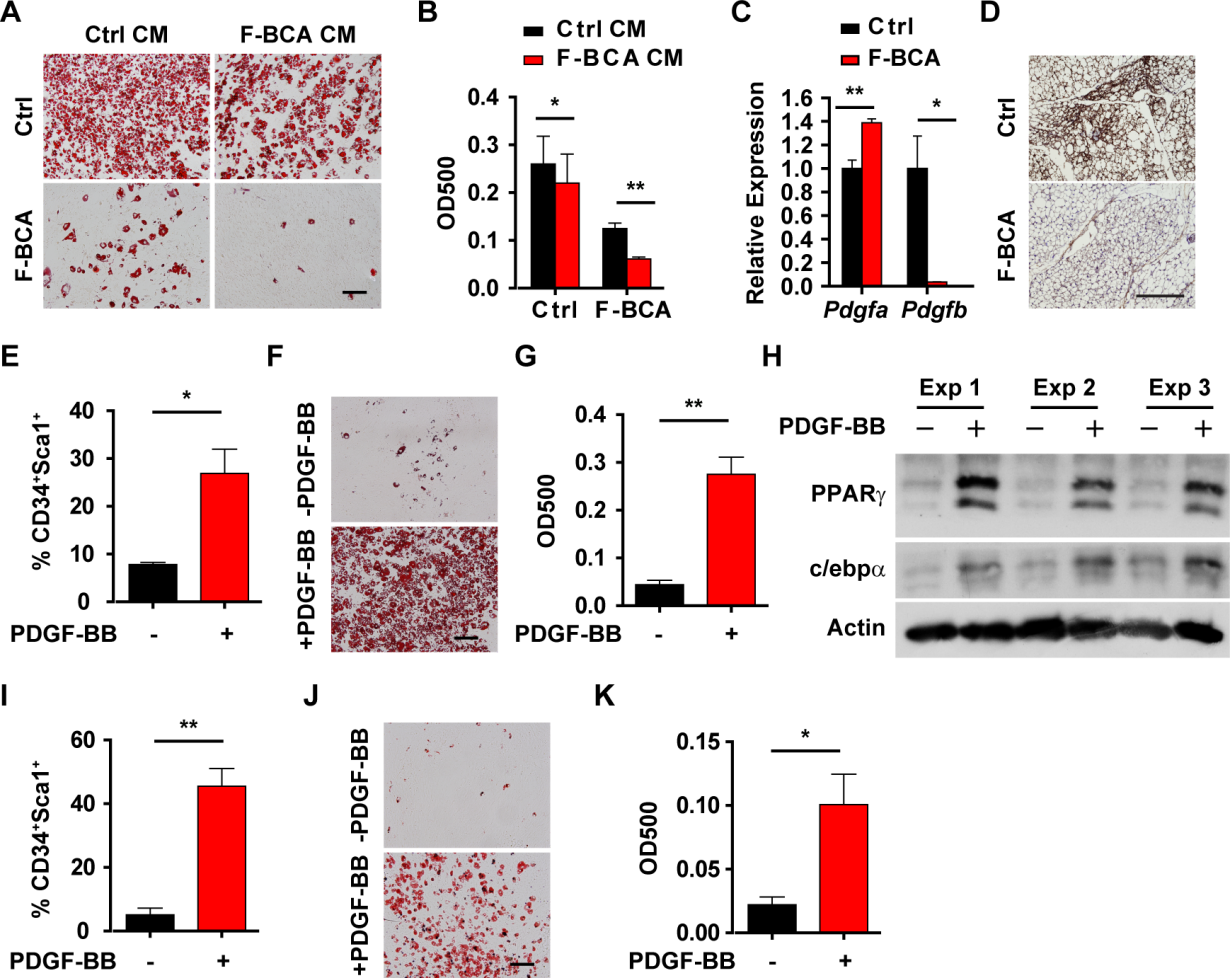
PDGF-BB is responsible for the maintenance of preadipocyte number. (A, B) Ctrl or F-BCA SVF cells were pretreated with CM harvested from Ctrl or F-BCA SVF cell culture before adipogenic induction. Adipogenesis was assayed with Oil Red O staining (panel A). Scale bar: 100 μm. Oil Red O staining was quantitated by isopropanol extraction (*n* = 3) (B). (C) RT-PCR analyses of PDGF expression in Ctrl and F-BCA SVF cells (*n* = 3). (D) Immunostaining of phospho-PDGFR-β on I-WAT sections from Ctrl and F-BCA mice at 4 months of age. Scale bar: 200 μm. (E) SVF cells isolated from F-BCA I-WAT were treated with 10 ng/mL PDGF-BB for 4 days before FACS analyses of CD34^+^Sca1^+^ populations (*n* = 4). (F, G) SVF cells isolated from F-BCA I-WAT were treated with 10 ng/mL PDGF-BB for 4 days and subjected to adipogenic induction. Adipogenesis was assayed with Oil Red O staining (panel F). Scale bar: 100 μm. Oil Red O staining was quantitated by isopropanol extraction (*n* = 6) (G). (H) Western blot analyses of PPARγ and c/ebpα in F-BCA SVF cells treated with or without 10 ng/mL PDGF-BB and subjected to adipogenic induction. (I–K) Matrigel plugs containing vehicle or 10 ng PDGF-BB were implanted in contralateral I-WATs of F-BCA mice for 2 weeks. Isolated SVF cells were subjected to FACS analyses (*n* = 4) (panel I) and adipogenic induction (panel J and K). Adipogenesis was assayed with Oil Red O staining (panel J). Oil Red O staining was quantitated by isopropanol extraction (*n* = 3) (panel K). Data are presented as mean ± SEM. Statistical analyses were performed with two-tailed unpaired (panel C) or paired (panel E, G, I, and K) student *t* test or two-way ANOVA followed by Bonferroni's multiple comparison test (panel B). *p < 0.05; **p < 0.01. Underlying data can be found in S1 Data. CD34, cluster of differentiation 34; CM, conditioned medium; Ctrl, control; FACS, fluorescence-activated cell sorting; F-BCA, *Fsp1*-*Cre*;*Ctnnb1*^exon 3 fl/+^; FSP1, fibroblast-specific protein-1; I-WAT, inguinal white adipose tissue; PDGF, platelet-derived growth factor; PDGFR-β, PDGF receptor-β; PPARγ, peroxisome proliferator-activated receptor-γ; RT-PCR, reverse transcription PCR; Sca1, stem cell antigen 1; SVF, stromal vascular fraction.

Conditioned medium from control SVF cells promoted F-BCA SVF cell differentiation (Fig 5A and 5B), indicating that secreted factors regulated preadipocyte differentiation. Expression of PDGFB was drastically decreased in the F-BCA SVF cells compared with that in the control SVF cells (Fig 5C). Immunohistochemical staining on the control and F-BCA WAT sections indicated reduced PDGFR-β phosphorylation in the F-BCA WATs (Fig 5D). Preadipocytes express PDGF receptors [2,5,6]. We next studied whether reduced PDGF expression is responsible for the loss of preadipocytes in the F-BCA SVFs. Treatment of the F-BCA SVF cells with PDGF-BB significantly increased the number of preadipocytes and restored the adipogenic potential (Fig 5E–5H). To study whether PDGF-BB is responsible for preadipocyte pool maintenance in vivo, growth factor-reduced Matrigel supplemented with PDGF-BB was implanted into the I-WATs of the F-BCA mice. Matrigel alone minimally affected the number and the adipogenic potential of the F-BCA SVF cells (Fig 5I–5K), whereas PDGF-BB containing Matrigel in the contralateral I-WAT significantly increased the percentage of preadipocytes and restored the adipogenic potential of the F-BCA SVF cells (Fig 5I–5K).

### FSP1^+^ fibroblasts regulate adipogenesis through extracellular matrix remodeling and YAP activation

**Fig 6:**
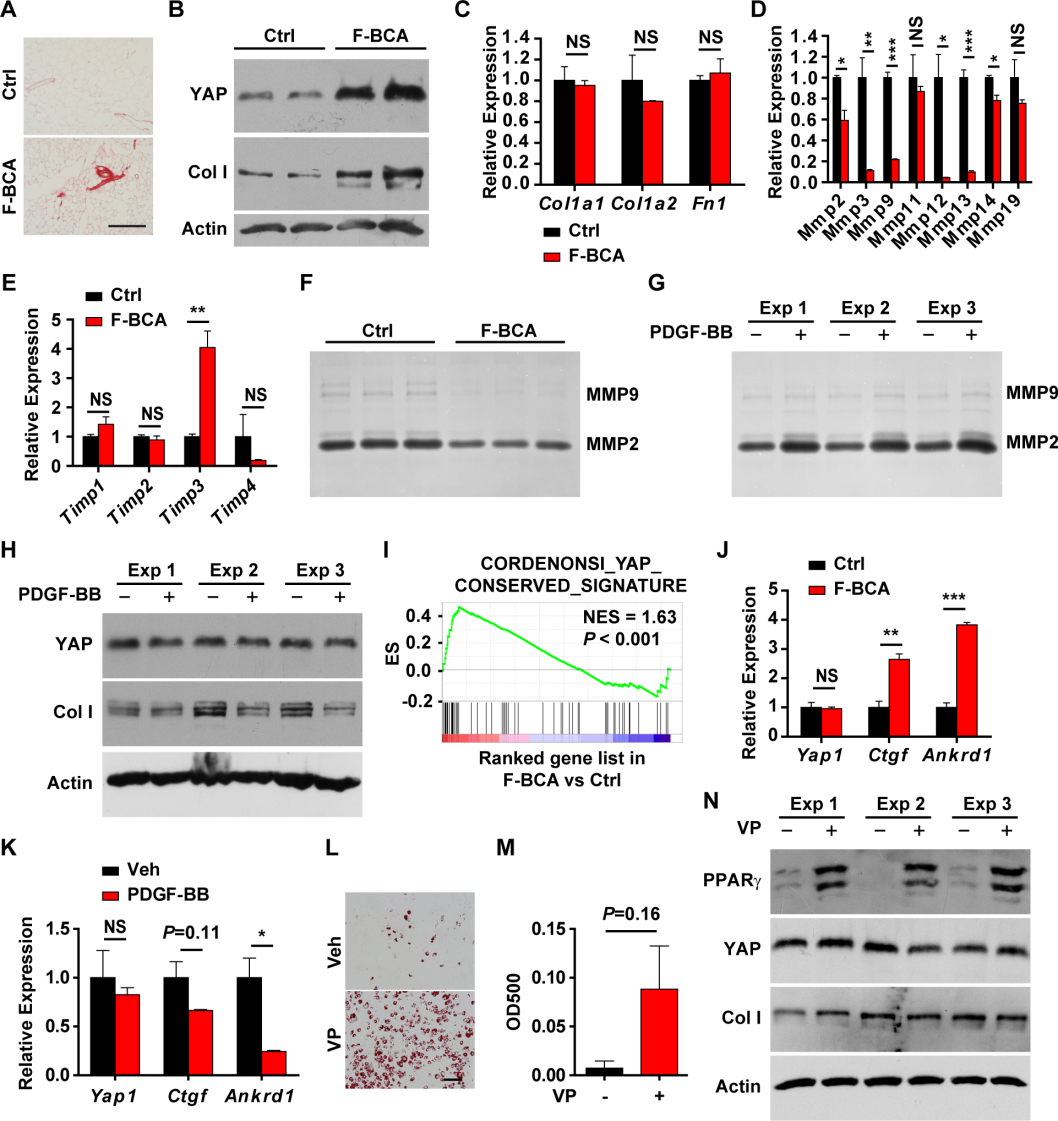
Activation of canonical Wnt signaling in FSP1^+^ fibroblasts modulates adipogenesis through extracellular matrix remodeling and YAP signaling. (A) Sirius red staining of WAT of 4-month-old F-BCA mice and their littermates. Scale bar: 200 μm. (B) Western blot analyses of Col I and YAP in control and F-BCA SVF cells. (C–E) RT-PCR analyses of expression of extracellular matrix proteins (panel C), MMPs (panel D), and TIMPs (panel E) in F-BCA and control SVF cells (*n* = 3). (F) Gelatin zymography of conditioned medium of F-BCA and control SVF cells. (G) Gelatin zymography of conditioned medium of F-BCA SVF cells treated with or without 10 ng/mL PDGF-BB. (H) Western blot analyses of Col I and YAP in F-BCA SVF cells treated with or without 10 ng/mL PDGF-BB. (I) GSEA of YAP signature in F-BCA SVF cells. (J) RT-PCR analyses of YAP and YAP target gene expression in F-BCA and control SVF cells (*n* = 3). (K) RT-PCR analyses of YAP and YAP target gene expression in F-BCA SVF cells treated with or without 10 ng/mL PDGF-BB (*n* = 3). (L, M) F-BCA SVF cells were pretreated with or without YAP inhibitor VP before adipogenic induction. Adipogenesis was assayed with Oil Red O staining (panel L). Scale bar: 100 μm. Oil Red O staining was quantitated by isopropanol extraction (*n* = 3) (panel M). (N) Western blot analyses of PPARγ, Col I, and YAP in F-BCA SVF cells treated with or without VP and subjected to adipogenic induction. Data are presented as mean ± SEM. Statistical analyses were performed with two-tailed unpaired (panel C, D, E, and J) or paired (panel K and M) student *t* test. *p < 0.05; **p < 0.01; ***p < 0.001. Underlying data can be found in S1 Data. Col I, type I collagen; F-BCA, *Fsp1*-*Cre*;*Ctnnb1*^exon 3 fl/+^; FSP1, fibroblast-specific protein-1; GSEA, gene set enrichment analysis; MMP, matrix metalloproteinase; NES, normalized enrichment score; NS, not significant; PDGF, platelet-derived growth factor; PPARγ, peroxisome proliferator-activated receptor-γ; RT-PCR, reverse transcription PCR; SVF, stromal vascular fraction; TIMP, tissue inhibitor of metalloproteinase; VP, verteporfin; WAT, white adipose tissue.

Activation of Wnt signaling in fibroblasts promotes tissue fibrosis [18–20]. Sirius red staining revealed significantly more collagen deposition in the F-BCA adipose tissues, particularly around the SVF zones (Fig 6A). More abundant type I collagen (Col I) expression was detected in the F-BCA SVF cells (Fig 6B), although the mRNA levels were unchanged upon activation of canonical Wnt signaling in the FSP1^+^ fibroblasts (Fig 6C). The matrix metalloproteinase (MMP) family is primarily responsible for the degradation and remodeling of extracellular matrix [6,21]. Proteolytic activity of MMPs is regulated by the tissue inhibitors of metalloproteinase (TIMPs) [6,21]. MMPs and TIMPs are differentially expressed in the adipose tissues during obesity [22] and modulate adipocyte differentiation [22–26]. Inhibition of MMP activity diminished the adipogenesis capability of preadipocytes [22]. F-BCA SVF cells had much reduced MMP expression and up-regulated TIMP-3 expression (Fig 6D and 6E). Gelatin zymography revealed significantly lower MMP expression in the F-BCA cells (Fig 6F). PDGF-BB treatment increased MMP expression and decreased TIMP3 expression (Fig 6G, S6A and S6B Fig). Despite the fact that the mRNA levels of collagen were not changed, Col I protein levels decreased upon PDGF-BB treatment (Fig 6H and S6C Fig). Excess collagen deposition and a stiff microenvironment inhibit preadipocyte differentiation [25–28]. Hippo pathway transcription factors YAP and TAZ are the major effectors sensing the mechanical signals exerted by extracellular matrix (ECM) physical property [28–32] that inhibits adipogenesis of mesenchymal stem cells [26,28,33]. Indeed, YAP protein accumulated in the F-BCA SVF cells (Fig 6B). The YAP signature was significantly enriched, and expression of YAP target genes CTGF and ANKRD1 was significantly up-regulated in the F-BCA SVF cells (Fig 6I and 6J). PDGF-BB treatment reduced the expression of YAP and its target genes (Fig 6H and 6K). To study whether YAP activation is responsible for the impaired adipogenic potential of F-BCA SVF cells, F-BCA SVF cells were pretreated with YAP pharmacological inhibitor verteporfin (VP) [34]. Inhibition of YAP signaling with VP restored the adipogenic potential of the F-BCA SVF cells, without affected Col I and MMP expression (Fig 6L–6N and S6D–S6G Fig). VP treatment did not increase the percentage of preadipocytes, suggesting that YAP mainly regulated differentiation capability (S6H Fig).

### Ablation of FSP1^+^ fibroblasts disturbs adipose homeostasis

**Fig 7:**
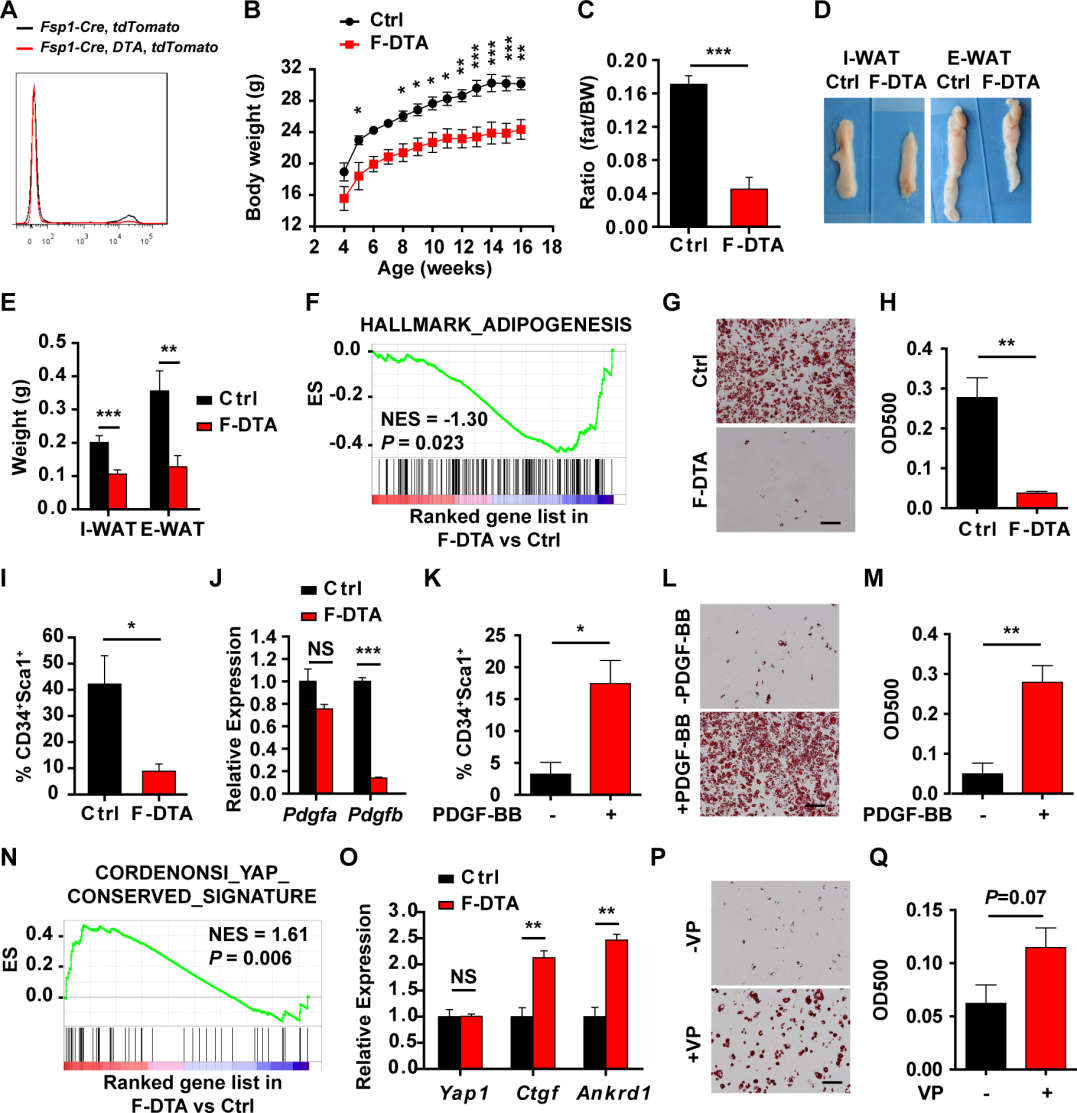
Ablation of FSP1^+^ fibroblasts disturbs adipose homeostasis. (A) FACS analyses of tdTomato^+^ cells in the SVF from *Fsp1*-*Cre*;*tdTomato* and *Fsp1*-*Cre*;*Rosa26*-*DTA*;*tdTomato* compound mice. (B) Body weight of male F-DTA compound mice and their littermates. *n* = 5 for male Ctrl mice; *n* = 5 for male F-DTA mice. (C) Percent body fat (NMR spectroscopy) of Ctrl and F-DTA littermates at 4 months of age. *n* = 9 for male Ctrl mice, and *n* = 8 for male F-DTA mice. (D, E) Representative pictures (panel D) and weight (panel E) of the adipose tissues of F-DTA mice and their littermates at 4 months of age. *n* = 12 for male male Ctrl mice, and *n* = 14 for male F-DTA mice. (F) GSEA data showing negative enrichment of adipogenesis signature in F-DTA SVF cells compared with the control SVF cells. (G, H) Control or F-DTA SVF cells were subjected to adipogenic induction. Adipogenesis was assayed with Oil Red O staining (panel G). Scale bar: 100 μm. Oil Red O staining was quantitated by isopropanol extraction (*n* = 3) (panel H). (I) FACS analyses of CD34^+^Sca1^+^ cells in the SVF of 4-month-old Ctrl and F-DTA mice (*n* = 3). (J) RT-PCR analyses of PDGF expression in control and F-DTA SVF cells (*n* = 3). (K) SVF cells isolated from F-DTA I-WAT were treated with 10 ng/mL PDGF-BB for 4 days before FACS analyses of CD34^+^Sca1^+^ populations (*n* = 3). (L, M) SVF cells isolated from F-DTA I-WAT were treated with 10 ng/mL PDGF-BB for 4 days and subjected to adipogenic induction. Adipogenesis was assayed with Oil Red O staining (panel L). Scale bar: 100 μm. Oil Red O staining was quantitated by isopropanol extraction (*n* = 3) (panel M). (N) GSEA of YAP signature in F-DTA SVF cells. (O) RT-PCR analyses of YAP and YAP target gene expression in F-DTA and control SVF cells (*n* = 3). (P, Q) F-DTA SVF cells were pretreated with or without YAP inhibitor VP before adipogenic induction. Adipogenesis was assayed with Oil Red O staining (panel P). Scale bar: 100 μm. Oil Red O staining was quantitated by isopropanol extraction (*n* = 3) (Q). Data are presented as mean ± SEM. Statistical analyses were performed with two-tailed unpaired (panel C, E, H, I, J, and O) or paired (panel K, M, and Q) student *t* test or two-way ANOVA followed by Bonferroni's multiple comparison test (panel B). *p < 0.05; **p < 0.01; ***p < 0.001. Underlying data can be found in S1 Data. CD34, cluster of differentiation 34; Ctrl, control; E-WAT, epididymal white adipose tissue; FACS, fluorescence-activated cell sorting; F-DTA, *Fsp1*-*Cre*;*Rosa26*-*DTA*; FSP1, fibroblast-specific protein-1; GSEA, gene set enrichment analysis; I-WAT, inguinal white adipose tissue; NES, normalized enrichment score; NMR, nuclear magnetic resonance; NS, not significant; PDGF, platelet-derived growth factor; RT-PCR, reverse transcription PCR; Sca1, stem cell antigen 1; SVF, stromal vascular fraction; VP, verteporfin.

To investigate the physiological requirement of the FSP1^+^ fibroblasts in the adipose development, FSP1^+^ fibroblasts were ablated by generating the *Fsp1*-*Cre*;*Rosa26*-*DTA* (F-DTA) compound mice (Fig 7A). At 4 months of age, F-DTA mice were significantly lean, with markedly reduced fat compared with the control mice (WT, *Fsp1*-*Cre*, or *Rosa26*-*DTA*) (Fig 7B and 7C, S7A and S7B Fig). Dissected WATs were smaller in size and weighed less in the F-DTA mice (Fig 7D and 7E). F-DTA male mice had significantly more food uptake, similar respiratory exchange ratio and physical activity, and slightly reduced energy expenditure compared with the control mice (S8A–S8D Fig), whereas F-DTA female mice had comparable food uptake, respiratory exchange ratio and physical activity, and slightly increased energy expenditure (S7C–S7F Fig). F-DTA mice more efficiently cleared glucose, but retained the same responsiveness to insulin, similar to that of the F-BCA mice (S8E–S8H Fig). No ectopic lipid deposition was observed in the livers of the F-DTA mice (S8I–S8K Fig). F-DTA SVF cells were defective in adipogenesis (Fig 7F–7H). F-DTA SVF cells contained much fewer preadipocytes (Fig 7I). PDGFB expression decreased in the F-DTA SVF cells (Fig 7J). PDGF-BB treatment increased the number of preadipocytes and restored the adipogenic potential of F-DTA SVF cells (Fig 7K–7M). Similar to the F-BCA SVF cells, F-DTA SVF cells had much reduced MMP expression, up-regulated TIMP-1 and TIMP-3 expression, and YAP activation (S9 Fig, Fig 7N and 7O). Inhibition of YAP signaling restored the adipogenic potential of the F-DTA SVF cells (Fig 7P and 7Q).

## Discussion

Adipocytes continuously turn over in adults. Like other adult stem cells and progenitor cells, preadipocytes are resident in a highly specialized niche. In this report, we identified FSP1^+^ fibroblasts as the niche for preadipocytes. FSP1^+^ fibroblasts are crucial to the maintenance of adipose homeostasis. Alteration in FSP1^+^ fibroblasts disturbs adipose homeostasis and results in loss of adiposity.

Preadipocytes rapidly expand from the preexisting pool during the first postnatal month [3]. During adulthood, preadipocytes and adipocytes are constantly renewed [35,36]. Turnover of preadipocytes and adipocytes is observed in both humans and mice [35,36]. Obese mice have increased adipocyte formation [35,36]. Adipogenic niches, including macrophage-related chronic inflammation [11,12], may have predominant roles in regulating the turnover of preadipocytes and adipocytes during adulthood and obesity. Numbers of FSP1^+^ fibroblasts increase during obesity. FSP1^+^ fibroblasts regulate adipose homeostasis by maintaining the preadipocyte pool and its differentiation potential. Alterations in FSP1^+^ fibroblasts result in diminished adipose depots at adulthood but not at puberty. Therefore, FSP1^+^ fibroblasts may represent a class of microenvironmental cues in regulating the turnover of preadipocytes and adipocytes and adipose homeostasis during adulthood and obesity. FSP1 is broadly expressed in mesenchymal cells. It should be noted that fibroblasts are highly heterogeneous. The heterogeneity of fibroblasts are reflected not only by the marker expression but also their biological functions. Subtypes of fibroblasts, similar to those of macrophages, were proposed [13]. Obesity induces macrophage polarization in adipose tissues [12]. It warrants further investigation into whether FSP1^+^ fibroblasts undergo similar polarization in obesity and whether subpopulations of FSP1^+^ fibroblasts regulate preadipocyte renewal and maintenance of adipose homeostasis.

Conditioned medium from the control SVF cells can restore the differentiation potential of the F-BCA SVF cells, suggesting that FSP1^+^ fibroblasts regulate preadipocyte renewal and adipose homeostasis via soluble factors. Alterations in FSP1^+^ fibroblasts greatly reduce PDGF-BB expression. PDGF-BB can restore preadipocyte numbers both in vitro and in vivo. In addition to the maintenance of the preadipocyte population, PDGF-BB also regulates adipogenic differentiation capability of the preadipocytes. Preadipocytes express PDGF receptors [2,5,6]. PDGF receptor expression in preadipocytes not only reflects the mesenchymal origin of the preadipocytes but may also play a central role in adipose homeostasis by maintaining both the preadipocyte pool and its adipogenic potential. The regulation of maintenance of the preadipocyte population and adipogenic potential by PDGF-BB may be exerted through distinct signaling pathways. Alterations in FSP1^+^ fibroblasts disturb extracellular matrix remodeling and YAP signaling in the adipose tissue microenvironment. PDGF-BB treatment restores extracellular matrix remodeling and alleviates YAP activation. However, inhibition of YAP signaling restores the adipogenic capability of the SVF cells but does not affect the number of preadipocytes.

It remains to be elucidated whether and how FSP1^+^ fibroblasts are regulated during adult adipose homeostasis. Upon high-fat–induced obesity, β-catenin target gene expression was altered in the FSP1^+^ fibroblasts. β-catenin-dependent canonical Wnt signaling is inhibitory to adipogenesis [5,8]. Wnt ligands produced by the preadipocytes, in particular Wnt10B, negatively regulate adipose homeostasis. Transgenic mice expressing Wnt10B showed a decrease in adipose mass [9]. Such a phenotype is largely attributed to activation of β-catenin in the preadipocytes [8,9]. Mice with β-catenin activation in mature adipocytes had normal adipose depots and metabolism, whereas activation of canonical Wnt signaling in PPARγ^+^ preadipocytes resulted in severe loss of adiposity [8,9], suggesting autocrine regulation of preadipocyte-origin Wnt ligands. However, Wnt ligands produced by the preadipocytes may also regulate adipose homeostasis via paracrine mechanisms. Activation of canonical Wnt signaling in the FSP1^+^ fibroblasts results in loss of adiposity and beneficial metabolism, similar to that observed in the mice with canonical Wnt signaling activation in the preadipocytes. Thus, Wnt ligands secreted by the preadipocytes may regulate adipose homeostasis via both autocrine and paracrine mechanisms orchestrating the functions of preadipocytes and their microenvironment.

In summary, FSP1^+^ fibroblasts are essential to the maintenance of the pool and differentiation potential of preadipocytes via PDGF signaling, extracellular matrix remodeling, and YAP activation. FSP1^+^ fibroblasts are a niche for preadipocytes orchestrating the functions of preadipocytes and their microenvironment.

## Materials and methods

### Ethics statement

All mice were housed in a specific pathogen-free environment at the Shanghai Institute of Biochemistry and Cell Biology and treated in strict accordance with protocols approved by the Institutional Animal Care and Use Committee of Shanghai Institute of Biochemistry and Cell Biology (approval number: NAF-15-003-S325-006).

### Mice

*Fsp1*-*Cre* mice [15] were from the Jackson Laboratory. *β*-*cat exon3*^*flox*/*+*^ mice were a generous gift from Professor Lijian Hui (Shanghai Institute of Biochemistry and Cell Biology). *Fsp1*-*Cre* and *β*-*cat exon3*^*flox*/*+*^ mice were backcrossed to the *FVB* background for >9 generations. *Rosa26*-*tdTomato*, *Rosa26*-*mTmG*, and *Rosa26*-*DTA* mice at C57Bl/6 and 129 mixed background were generous gifts from Professor Yi Zeng (Shanghai Institute of Biochemistry and Cell Biology). For diet-induced obesity studies, starting at 4 weeks of age, mice were fed with HFDs containing 60% kcal from fat for 12 weeks (Research Diets, New Brunswick, New Jersey).

### Glucose tolerance tests and insulin tolerance tests

Glucose tolerance tests (GTTs) and insulin tolerance tests (ITTs) were performed as described [37]. For GTTs, mice were fasted overnight and received an intraperitoneal injection of 2 g/kg body weight glucose (Sigma-Aldrich, St. Louis, Missouri). For ITTs, mice were injected intraperitoneally with 0.5 U/kg body weight insulin (Biosynthetic Human Insulin, 100 U/mL; Novo Nordisk, Bagsvaerd, Denmark) after a 4-hour fast. Tail blood glucose levels were measured at 0, 15, 30, 60, 90, and 120 minutes after challenge using the Onetouch Ultra blood glucose monitoring system (LifeScan, Shanghai, China). AUC was calculated using GraphPad Prism.

### Metabolic measurements

Metabolic measurements with indirect calorimetry were performed on mice as described [38]. Animals were maintained on a ND or an HFD at ambient temperature under 12-hour light and dark cycles. Mice were acclimated in a comprehensive lab animal monitoring system (Columbus Instruments, Columbus, Ohio) for 1 day before recording over 2 days following the manufacturer's instruction.

### Hematoxylin–eosin and immunofluorescent staining

Mouse WATs were isolated and fixed in 4% PFA followed by embedding in paraffin. Paraffin-embedded tissues were sectioned and stained with hematoxylin–eosin (HE). The immunostaining was performed as previously described [39]. Sections were incubated at 4°C overnight with primary antibodies. Histology or immunostained samples were viewed under microscope (IX71; OLYMPUS, Tokyo, Japan) with a UPlan-FLN 4× objective/0.13 PhL, a UPlan-FLN 10× objective/0.30 PhL, or a LUCPlan-FLN 20× objective/0.45 PhL. Images were captured with a digital camera (IX-SPT; OLYMPUS, Tokyo, Japan) and Digital Acquire software (DPController; OLYMPUS, Tokyo, Japan). WATs were viewed under microscope (SZX16; OLYMPUS, Tokyo, Japan) with an SDF-PLAPO 1× PF. Images were captured with a digital camera (U-LH100HGAPO; OLYMPUS, Tokyo, Japan) and Digital Acquire software (DPController; OLYMPUS, Tokyo, Japan).

### SVF cell isolation and culture

I-WAT and E-WAT were washed with PBS, cut into small pieces, and digested with 1 mg/mL Collagenase (Worthington, Lakewood, New Jersey) at 37°C for 60 minutes. SVF cell culture, FACS analyses, and adipogenic induction were performed as described [40,41]. For PDGF and VP treatment experiments, SVF cells were treated with PDGF or VP for 4 days before FACS analyses and adipogenic induction. To isolate floated adipocytes, collagenase-treated adipose depot mixture was centrifuged at 200 g for 1 minute. Floating cell layer was collected as adipocytes.

### Western blot analysis

Western blot analysis was performed as previously described [39].

### Quantitative RT-PCR

Total RNA was prepared from SVF cells using Trizol reagents (Invitrogen) as previously described [39]. Equal amounts of RNA were subjected to quantitative RT-PCR using SYBR green with the BIO-RAD Q-PCR Systems according to the manufacturer's protocol. Relative expression levels were calculated using the comparative CT method. Gene expression levels were normalized to *Actin*. The primers used are listed in S1 Table.

### RNA-Seq, GO analysis, and GSEA

SVF cells were isolated from pooled WATs from control, F-BCA, and F-DTA mice. Total RNA was extracted and purified with TRIzol. Two biological replicates were subjected to complementary DNA library preparation and sequencing according to the Illumina standard protocol. RNA-seq reads were mapped to mm9 reference genome using TopHat2. Mapped reads of the 2 replicates in each group were merged together for further analysis. Expression for each known gene from RefSeq was determined by covered reads and normalized with RPKM. Normalized gene expression of the SVF cells are listed in S2 Table. Differentially expressed genes (DEGs) were identified by gene expression comparison between control and F-BCA or F-DTA SVF cells and were defined with parameters including *p* < 0.01 (Wald test), fold change ≥ 1.5 or ≤ 0.667, and expression level ≥ 5 RPKM in at least one sample. Gene ontology (GO) analysis was performed with the DAVID online tool. Top GO categories were selected according to the *p*-value after Benjamini-Hochberg correction. GSEA was performed on gene signatures obtained from the MSigDB database version 5.0 (March 2015 release) [42]. Statistical significance was assessed by comparing the enrichment score to enrichment results generated from 1,000 random permutations of the gene set to obtain *p*-values (nominal *p*-value).

### Statistics

Sample sizes for each figure are denoted in the figure legends. Data are representative of at least 3 biologically independent experiments. For animal experiments, the sample size is determined on the basis of our prior knowledge of the variability of experimental output. Age of animals was matched. The experiments were not randomized, and the investigators were not blinded to allocation during experiments and outcome assessment. Statistical significance between conditions was assessed by unpaired or paired two-tailed student *t* tests or ANOVAs followed by Bonferroni's multiple comparison test. All error bars represent SEM, and significance between conditions is denoted *p < 0.05; **p < 0.01; and *p < 0.001. “NS” denotes not significant. The numerical data used in all figures are included in S1 Data.

## Supporting information

S1 Fig FSP1^+^ cells in the SVF express fibroblastic markers. (A) Whole mount images of I-WAT and E-WAT and tail of 4-month-old *tdTomato* or *Fsp1*-*Cre*;*tdTomato* mice. No tdTomato signal was detected in the WAT of *Fsp1*-*Cre*;*tdTomato* mice. Scale bar: 1 mm. (B, C) Tail tip fibroblasts (panel B) and SVF cells (panel C) isolated from I-WAT of *Fsp1*-*Cre*;*tdTomato* mice were stained with anti-αSMA or Vim antibody and subjected to flow cytometry analysis. (D, E) *Fsp1*-*Cre*;*tdTomato* mice were fed with an HFD for 12 weeks to induce obesity. SVF cells isolated from I-WAT were stained with anti-αSMA or Vim (panel D) and CD34 and Sca1 (panel E) antibodies and subjected to flow cytometry analysis. αSMA, α-smooth muscle actin; CD34, cluster of differentiation 34; E-WAT, epididymal white adipose tissue; FSP1, fibroblast-specific protein-1; HFD, high-fat diet; I-WAT, inguinal white adipose tissue; Sca1, stem cell antigen-1; SVF, stromal vascular fraction; Vim, vimentin.
(TIF)

S2 Fig FSP1^+^ cells in the SVF are not in the adipogenic lineage in the *Fsp1*-*Cre*;*Ctnnb1*^exon 3 fl/+^ compound mice. (A) Expression of β-catenin target genes in FACS-sorted tdTomato^+^ and tdTomato^−^ SVF cells isolated from I-WAT of *Fsp1*-*Cre*;*tdTomato* mice fed with ND or HFD. (B) Western blot analyses of β-catenin expression in I-WAT, E-WAT, adipocytes, and SVF cells isolated from WATs from F-BCA compound mice and their littermates. TTFs were used as a positive control. (C) Flow cytometry analysis of tdTomato^+^ cells in the *Fsp1*-*Cre*;*Ctnnb1*^exon 3 fl/+^;*tdTomato* compound mice. (D) RT-PCR analyses of β-catenin and its target gene *Axin2* in FACS-sorted tdTomato^+^ and tdTomato^−^ SVF cells isolated from I-WAT of *Fsp1*-*Cre*;*tdTomato* and *Fsp1*-*Cre*; *Ctnnb1*^exon 3 fl/+^;*tdTomato* mice. (E) Flow cytometry analysis of CD34^+^Sca1^+^ cells in the *Fsp1*-*Cre*;*Ctnnb1*^exon 3 fl/+^;*tdTomato* compound mice. CD34, cluster of differentiation 34; E-WAT, epididymal white adipose tissue; FACS, fluorescence-activated cell sorting; F-BCA, *Fsp1*-*Cre*;*Ctnnb1*^exon 3 fl/+^; FSP1, fibroblast-specific protein-1; HFD, high-fat diet; I-WAT, inguinal white adipose tissue; ND, normal-chow diet; RT-PCR, reverse transcription PCR; Sca1, stem cell antigen-1; SVF, stromal vascular fraction; TTF, tail tip fibroblast.
(TIF)

S3 Fig Activation of canonical Wnt signaling in FSP1^+^ fibroblasts results in gradual loss of adipose tissues. (A) Body weight of male F-BCA compound mice and their littermates at 3 weeks of age. *n* = 25 for male control mice; *n* = 23 for male F-BCA mice. (B) Ventral view of subcutaneous and visceral adipose depots of control and F-BCA littermates at 3 weeks of age. Adipose depots are circled with dashed lines. (C) Representative pictures of the adipose tissues of F-BCA mice and their littermates at 3 weeks of age. (D) HE staining of WAT of 3-week-old F-BCA mice and their littermates. Scale bar: 200 μm. (E) Body weight of male F-BCA compound mice and their littermates at 8 months of age. *n* = 11 for male control mice; *n* = 5 for male F-BCA mice. (F) Ventral view of subcutaneous and visceral adipose depots of control and F-BCA littermates at 8 months of age. Adipose depots are circled with dashed lines. (G) Representative pictures of the adipose tissues of F-BCA mice and their littermates at 8 months of age. (H) HE staining of WAT of 8-month-old F-BCA mice and their littermates. Scale bar: 200 μm. Data are presented as mean ± SEM. Statistical analyses were performed with two-tailed unpaired student *t* test. ***p < 0.001. Underlying data can be found in S1 Data. NS, not significant. F-BCA, *Fsp1*-*Cre*;*Ctnnb1*^exon 3 fl/+^; FSP1, fibroblast-specific protein-1; HE, hematoxylin–eosin; WAT, white adipose tissue.
(TIF)

S4 Fig Activation of canonical Wnt signaling in FSP1^+^ fibroblasts does not result in metabolic disorders associated with classical lipodystrophy. (A–H) Metabolic cage analyses were performed on F-BCA mice on ND (panel A–D) or on HFD (panel E–H). Food consumption (panel A and E), EE (panel B and F), RER (panel C and G), and XTOT (panel D and H) were recorded. *n* = 6 for each group. (I) Weight of liver, kidney, and testis of the F-BCA mice and their littermates at 4 months of age (liver: 13 Ctrl, 12 F-BCA; kidney: 6 Ctrl, 8 F-BCA; testis: 6 Ctrl, 6 F-BCA). (J) HE staining of liver sections of 4-month-old mice on ND or HFD. Scale bar: 200 μm. (K) Hepatic triglyceride levels in mice on ND or HFD. *n* = 5 for each group. Data are presented as mean ± SEM. Statistical analyses were performed with two-tailed unpaired student *t* test or one way ANOVA followed by Bonferroni's multiple comparison test. *p < 0.05; **p < 0.01; ***p < 0.001. Underlying data can be found in S1 Data. Ctrl, control; EE, energy expenditure; F-BCA, *Fsp1*-*Cre*;*Ctnnb1*^exon 3 fl/+^; FSP1, fibroblast-specific protein-1; HFD, high-fat diet; ND, normal-chow diet; NS, not significant; RER, respiratory exchange ratio; XTOT, physical activity.
(TIF)

S5 Fig Activation of canonical Wnt signaling in fibroblasts disturbs adipose homeostasis of female mice. (A) Body weight of female control and F-BCA mice on ND. *n* = 8 for female control mice, and *n* = 8 for female F-BCA mice. (B) Weight of the adipose tissues of female control and F-BCA mice on ND at 4 months of age (I-WAT: 5 Ctrl, 9 F-BCA; G-WAT: 4 Ctrl, 7 F-BCA). (C–F) Metabolic cage analyses were performed on female control and F-BCA mice on ND. Food consumption (panel C), EE (panel D), RER (panel E), and XTOT (panel F) were recorded. *n* = 7 for female control mice, and *n* = 5 for female F-BCA mice. Data are presented as mean ± SEM. Statistical analyses were performed with two-tailed unpaired student *t* test or two-way ANOVA followed by Bonferroni's multiple comparison test (panel A). *p < 0.05; **p < 0.01. Underlying data can be found in S1 Data. Ctrl, control; EE, energy expenditure; F-BCA, *Fsp1*-*Cre*;*Ctnnb1*^exon 3 fl/+^; FSP1, fibroblast-specific protein-1; G-WAT, gonadal white adipose tissue; I-WAT, inguinal white adipose tissue; ND, normal-chow diet; NS, not significant; RER, respiratory exchange ratio; XTOT, physical activity.
(TIF)

S6 Fig PDGF-BB regulates MMP expression in F-BCA SVF cells. (A–F) RT-PCR analyses of expression of extracellular matrix proteins (panel C and D), MMPs (panel A and E) and TIMPs (panel B and F) in F-BCA SVF cells treated with or without 10 ng/mL PDGF-BB (panel A and C) or 0.5 μg/mL VP (panel D and F) (*n* = 3). (G) Gelatin zymography of conditioned medium of F-BCA SVF cells treated with or without 0.5 μg/mL VP. (H) SVF cells isolated from F-BCA I-WAT were treated with 0.5 μg/mL VP for 4 days before FACS analyses of CD34^+^Sca1^+^ populations (*n* = 3). Data are presented as mean ± SEM. Statistical analyses were performed with two-tailed paired student *t* test. *p < 0.05; **p < 0.01. Underlying data can be found in S1 Data. CD34, cluster of differentiation 34; FACS, fluorescence-activated cell sorting; F-BCA, *Fsp1*-*Cre*;*Ctnnb1*^exon 3 fl/+^; FSP1, fibroblast-specific protein-1; I-WAT, inguinal white adipose tissue; MMP, matrix metalloproteinase; NS, not significant; PDGF, platelet-derived growth factor; Sca1, stem cell antigen-1; SVF, stromal vascular fraction; TIMP, tissue inhibitor of metalloproteinase; VP, verteporfin.
(TIF)

S7 Fig Ablation of FSP1^+^ fibroblasts disturbs adipose homeostasis of female mice. (A) Body weight of female control and F-DTA mice on ND. *n* = 7 for Ctrl mice and *n* = 7 for F-DTA mice. (B) Weight of the adipose tissues of female control and F-DTA mice on ND at 4 month age. *n* = 8 for female Ctrl mice, and *n* = 6 for female F-DTA mice. (C–F) Metabolic cage analyses were performed on control and F-DTA female mice on ND. Food consumption (panel C), EE (panel D), RER (panel E), and XTOT (panel F) were recorded. *n* = 6 for female Ctrl mice, except *n* = 7 for food consumption and *n* = 4 for female F-DTA mice. Data are presented as mean ± SEM. Statistical analyses were performed with two-tailed unpaired student *t* test (panel B–F) or two-way ANOVA followed by Bonferroni's multiple comparison test (panel A). *p < 0.05; **p < 0.01. Underlying data can be found in S1 Data. Ctrl, control; EE, energy expenditure; F-DTA, *Fsp1*-*Cre*;*Rosa26*-*DTA*; FSP1, fibroblast-specific protein-1; ND, normal-chow diet; NS, not significant; RER, respiratory exchange ratio; XTOT, physical activity.
(TIF)

S8 Fig Ablation of FSP1^+^ fibroblasts affects metabolism. (A–D) Metabolic cage analyses were performed on male control and F-DTA mice. Food consumption (panel A), EE (panel B), RER (panel C), and XTOT (panel D) were recorded. *n* = 6 for each group, except *n* = 5 for food consumption. (E) GTT of male control and F-DTA mice at 4 months of age. *n* = 14 for male control mice; *n* = 10 for male F-DTA mice. (F) Quantification of the AUC of the GTT in panel E. (G) ITT of male control and F-DTA mice at 4 months of age. *n* = 10 for male control mice; *n* = 6 for male F-DTA mice. (H) Quantification of the AUC of the ITT in panel G. (I) Weight of liver, kidney, and testis of the 4-month-old male control and F-DTA mice (liver: 10 Ctrl, 11 F-DTA; kidney: 6 Ctrl, 6 F-DTA; testis: 8 Ctrl, 8 F-DTA). (J) HE staining of liver sections of 4-month-old male control and F-DTA mice. Scale bar: 200 μm. (K) Hepatic triglyceride levels in 4-month-old male control and F-DTA mice. *n* = 5 for each group. Data are presented as mean ± SEM. Statistical analyses were performed with two-tailed unpaired student *t* test or two-way ANOVA followed by Bonferroni's multiple comparison test (panel E and G). *p < 0.05; **p < 0.01; ***p < 0.001. Underlying data can be found in S1 Data. AUC, area under the curve; EE, energy expenditure; F-DTA, *Fsp1*-*Cre*;*Rosa26*-*DTA*; FSP1, fibroblast-specific protein-1; GTT, glucose tolerance test; HE, hematoxylin–eosin; ITT, insulin tolerance test; NS, not significant; RER, respiratory exchange ratio; XTOT, physical activity.
(TIF)

S9 Fig Ablation of FSP1^+^ fibroblasts modulates adipogenesis through YAP signaling. (A–C) RT-PCR analyses of expression of extracellular matrix proteins (panel A), MMPs (panel B), and TIMPs (panel C) in F-DTA and control SVF cells (*n* = 3). (D) Gelatin zymography of conditioned medium of F-DTA and control SVF cells. (E) Gelatin zymography of conditioned medium of F-DTA treated with or without 10 ng/mL PDGF-BB or 0.5 μg/mL VP. Data are presented as mean ± SEM. Statistical analyses were performed with two-tailed unpaired student *t* test. *p < 0.05; **p < 0.01; ***p < 0.001. Underlying data can be found in S1 Data. F-DTA, *Fsp1*-*Cre*;*Rosa26*-*DTA*; FSP1, fibroblast-specific protein-1; MMP, matrix metalloproteinase; NS, not significant; PDGF, platelet-derived growth factor; RT-PCR, reverse transcription PCR; SVF, stromal vascular fraction; TIMP, tissue inhibitor of metalloproteinase; YAP, Yes-associated protein.
(TIF)

S1 Table Primers used for qRT-PCR. qRT-PCR, quantitative reverse transcription PCR.
(DOCX)

S2 Table Normalized gene expression in control, F-BCA, and F-DTA SVF cells. F-BCA, *Fsp1*-*Cre*;*Ctnnb1*^exon 3 fl/+^; F-DTA, *Fsp1*-*Cre*;*Rosa26*-*DTA*; SVF, stromal vascular fraction.
(XLSX)

S1 Data Excel spreadsheet containing the underlying numerical data and statistical analysis for Figs 2B, 2D, 2F, 2H, 2I, 2J, 2K; 3A, 3C, 3E, 3G, 3H, 3I, 3J; 4C, 4D, 4F, 4H; 5B, 5C, 5E, 5G, 5I, 5K; 6C, 6D, 6E, 6J, 6K, 6M; 7B, 7C, 7E, 7H, 7I, 7J, 7K, 7M, 7O, 7Q; S3A, S3E; S4A, S4B, S4C, S4D, S4E, S4F, S4G, S4H, S4I, S4K; S5A, S5B, S5C, S5D, S5E, S5F; S6A, S6B, S6C, S6D, S6E, S6F, S6H; S7A, S7B, S7C, S7D, S7E, S7F; S8A, S8B, S8C, S8D, S8E, S8F, S8G, S8H, S8I, S8K and S9A, S9B, S9C. (XLSX)

## Abbreviations

αSMA: α-smooth muscle actin
AUC: area under the curve
CD34: cluster of differentiation 34
Col I: type I collagen
DEG: differentially expressed gene
ECM: extracellular matrix
E-WAT: epididymal white adipose tissue
FACS: fluorescence-activated cell sorting
F-BCA: *Fsp1*-*Cre*;*Ctnnb1*^exon 3 fl/+^
F-DTA: *Fsp1*-*Cre*;*Rosa26*-*DTA*
F-mTmG: *Fsp1*-*Cre*;*Rosa26*-flox-membrane-targeted Tomato-flox-membrane-targeted GFP
FSP1: fibroblast-specific protein-1
GFP: green fluorescent protein
GO: gene ontology
GSEA: gene set enrichment analysis
GTT: glucose tolerance test
HE: hematoxylin–eosin
HFD: high-fat diet
ITT: insulin tolerance test
I-WAT: inguinal white adipose tissue
MMP: matrix metalloproteinase
NMR: nuclear magnetic resonance
ND: normal-chow diet
NES: normalized enrichment score
NG2: neural glial antigen 2
PDGF: platelet-derived growth factor
PDGFR-β: PDGF receptor-β
PPARγ: peroxisome proliferator-activated receptor-γ
RT-PCR: reverse transcription PCR
Sca1: stem cell antigen-1
SVF: stromal vascular fraction
TIMP: tissue inhibitor of metalloproteinase
VP: verteporfin
WAT: white adipose tissue
WT: wild-type
YAP: Yes-associated protein
