# Supplementary figures and images for: FSP1-positive fibroblasts are adipogenic niche and regulate adipose homeostasis
Source: PLoS Biol. 2018 Aug 6;16(8):e2001493. doi: 10.1371/journal.pbio.2001493 (PMC6078284; doi:10.1371/journal.pbio.2001493)

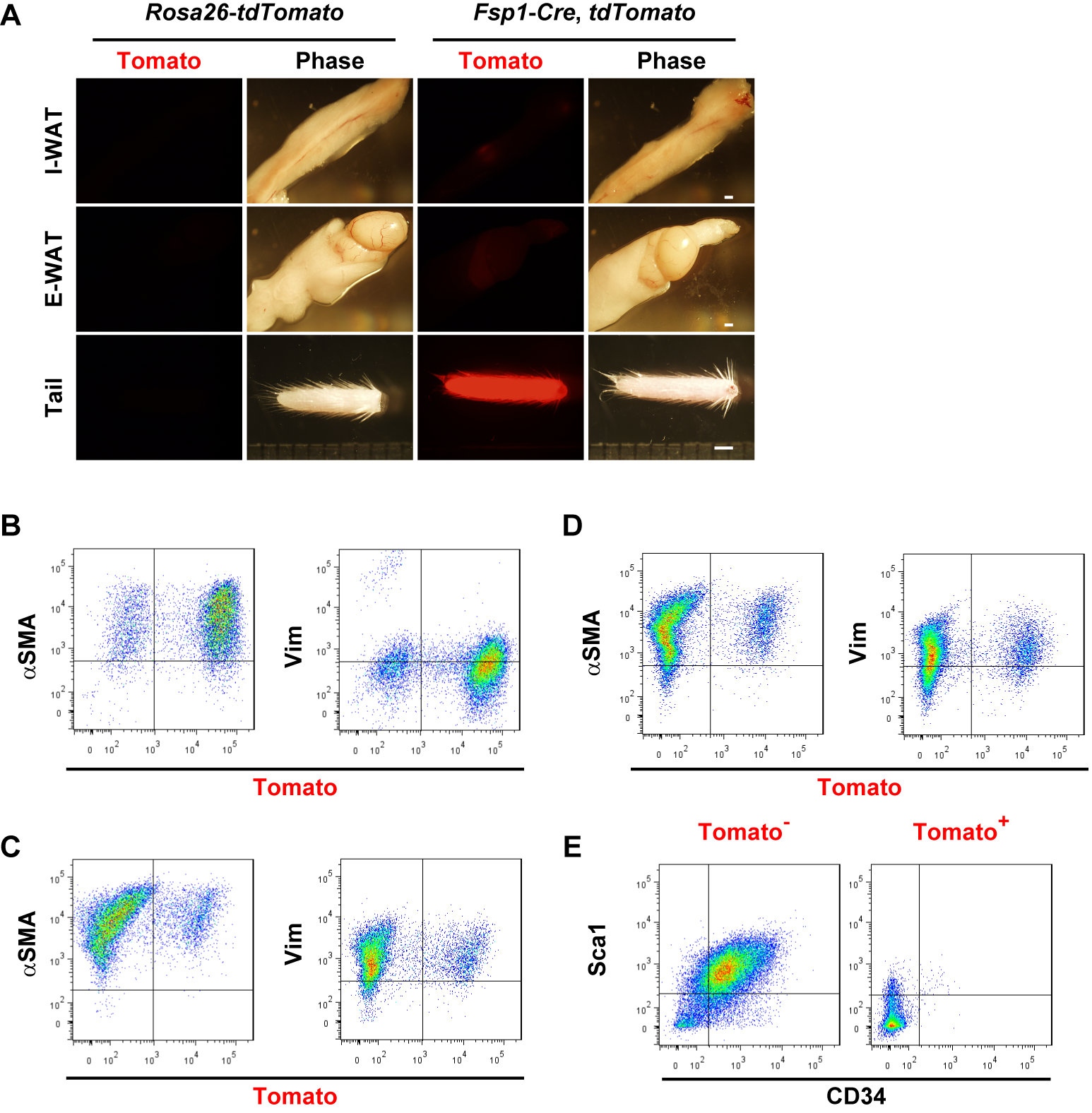

Supplement: S1 Fig — (A) Whole mount images of I-WAT and E-WAT and tail of 4-month-old tdTomato or Fsp1-Cre;tdTomato mice. No tdTomato signal was detected in the WAT of Fsp1-Cre;tdTomato mice. Scale bar: 1 mm. (B, C) Tail tip fibroblasts (panel B) and SVF cells (panel C) isolated from I-WAT of Fsp1-Cre;tdTomato mice were stained with anti-αSMA or Vim antibody and subjected to flow cytometry analysis. (D, E) Fsp1-Cre;tdTomato mice were fed with an HFD for 12 weeks to induce obesity. SVF cells isolated from I-WAT were stained with anti-αSMA or Vim (panel D) and CD34 and Sca1 (panel E) antibodies and subjected to flow cytometry analysis. αSMA, α-smooth muscle actin; CD34, cluster of differentiation 34; E-WAT, epididymal white adipose tissue; FSP1, fibroblast-specific protein-1; HFD, high-fat diet; I-WAT, inguinal white adipose tissue; Sca1, stem cell antigen-1; SVF, stromal vascular fraction; Vim, vimentin. (TIF) [file pbio.2001493.s001.tif]

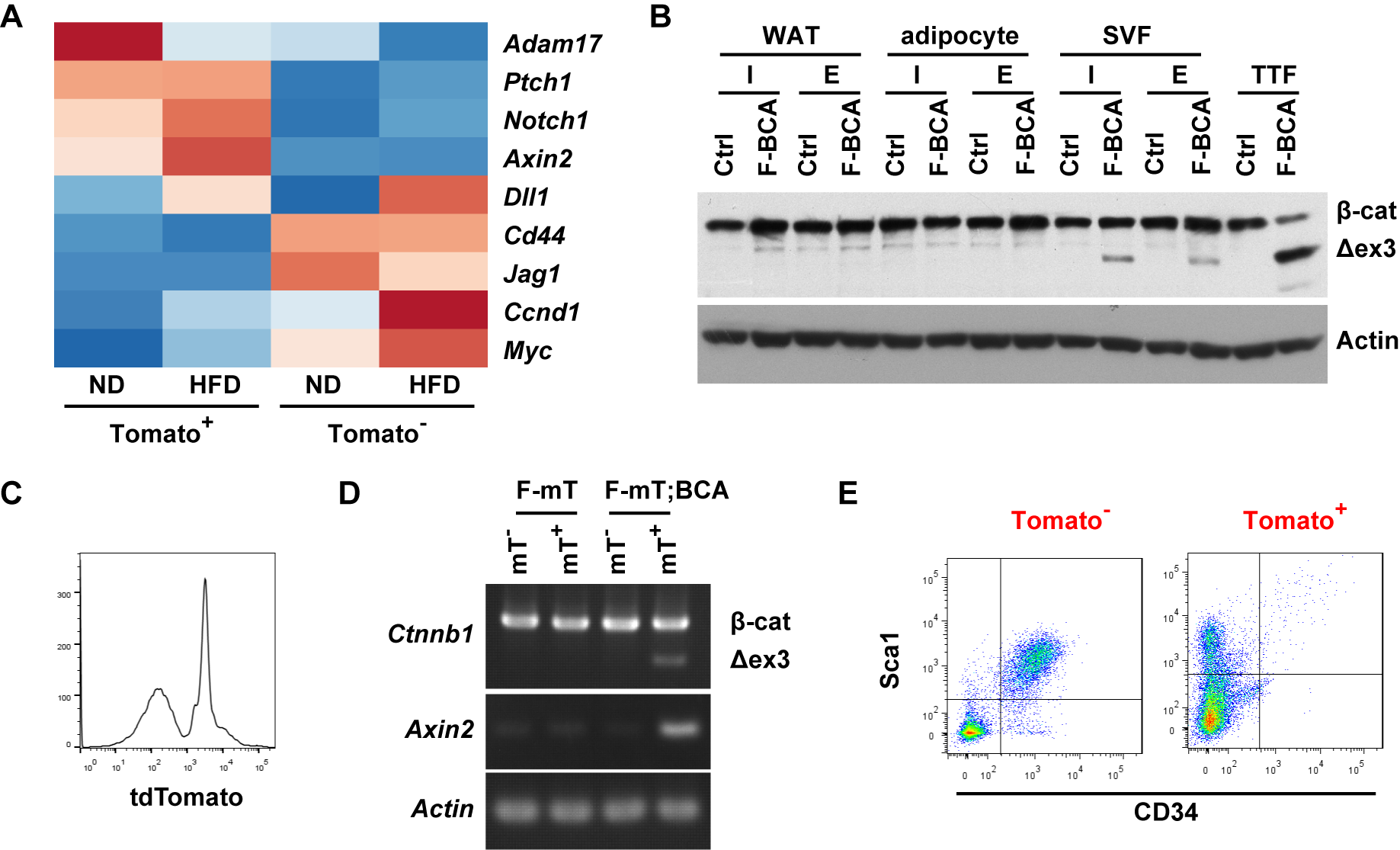

Supplement: S2 Fig — (A) Expression of β-catenin target genes in FACS-sorted tdTomato+ and tdTomato− SVF cells isolated from I-WAT of Fsp1-Cre;tdTomato mice fed with ND or HFD. (B) Western blot analyses of β-catenin expression in I-WAT, E-WAT, adipocytes, and SVF cells isolated from WATs from F-BCA compound mice and their littermates. TTFs were used as a positive control. (C) Flow cytometry analysis of tdTomato+ cells in the Fsp1-Cre;Ctnnb1exon 3 fl/+;tdTomato compound mice. (D) RT-PCR analyses of β-catenin and its target gene Axin2 in FACS-sorted tdTomato+ and tdTomato− SVF cells isolated from I-WAT of Fsp1-Cre;tdTomato and Fsp1-Cre; Ctnnb1exon 3 fl/+;tdTomato mice. (E) Flow cytometry analysis of CD34+Sca1+ cells in the Fsp1-Cre;Ctnnb1exon 3 fl/+;tdTomato compound mice. CD34, cluster of differentiation 34; E-WAT, epididymal white adipose tissue; FACS, fluorescence-activated cell sorting; F-BCA, Fsp1-Cre;Ctnnb1exon 3 fl/+; FSP1, fibroblast-specific protein-1; HFD, high-fat diet; I-WAT, inguinal white adipose tissue; ND, normal-chow diet; RT-PCR, reverse transcription PCR; Sca1, stem cell antigen-1; SVF, stromal vascular fraction; TTF, tail tip fibroblast. (TIF) [file pbio.2001493.s002.tif]

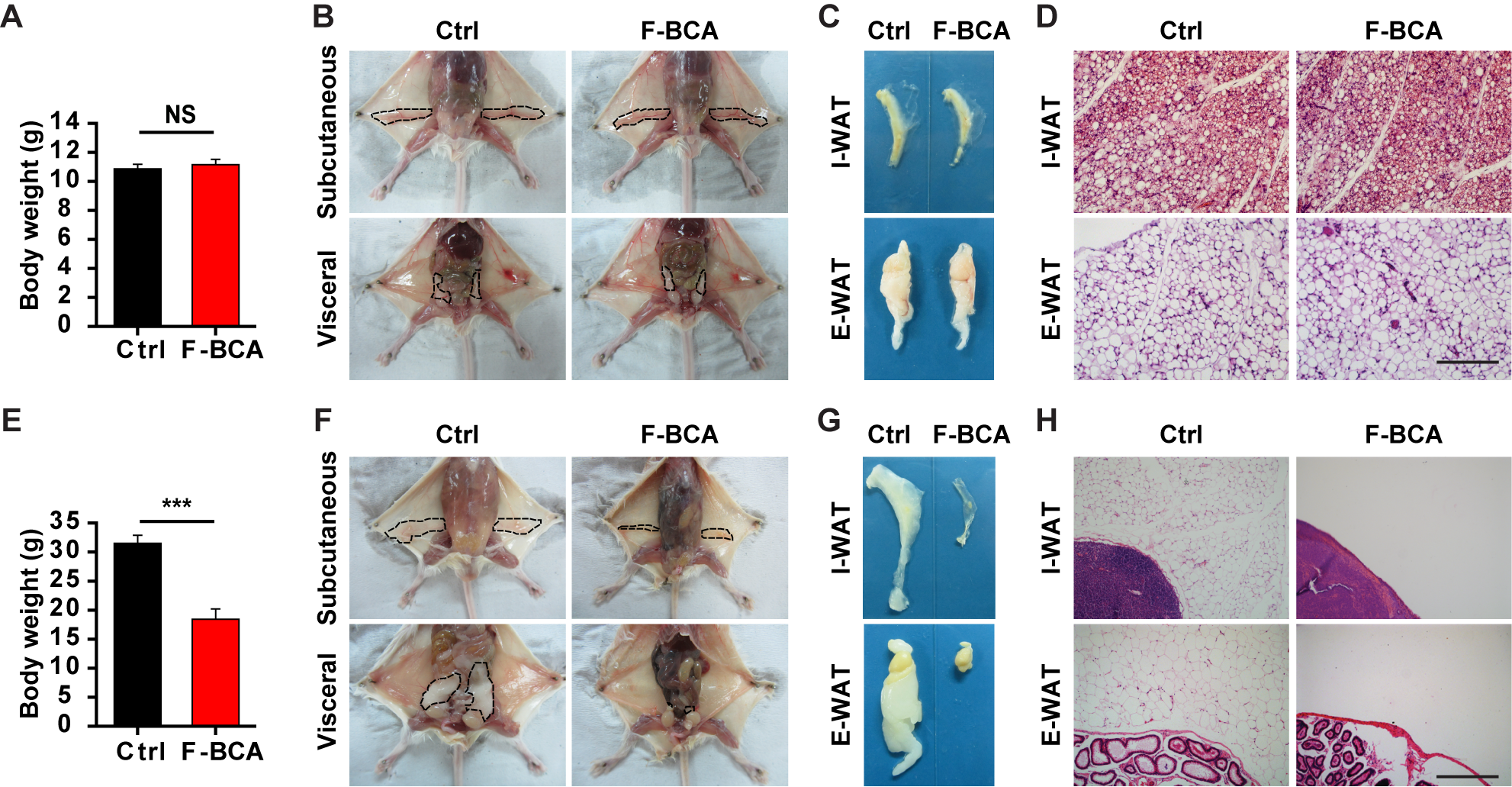

Supplement: S3 Fig — (A) Body weight of male F-BCA compound mice and their littermates at 3 weeks of age. n = 25 for male control mice; n = 23 for male F-BCA mice. (B) Ventral view of subcutaneous and visceral adipose depots of control and F-BCA littermates at 3 weeks of age. Adipose depots are circled with dashed lines. (C) Representative pictures of the adipose tissues of F-BCA mice and their littermates at 3 weeks of age. (D) HE staining of WAT of 3-week-old F-BCA mice and their littermates. Scale bar: 200 μm. (E) Body weight of male F-BCA compound mice and their littermates at 8 months of age. n = 11 for male control mice; n = 5 for male F-BCA mice. (F) Ventral view of subcutaneous and visceral adipose depots of control and F-BCA littermates at 8 months of age. Adipose depots are circled with dashed lines. (G) Representative pictures of the adipose tissues of F-BCA mice and their littermates at 8 months of age. (H) HE staining of WAT of 8-month-old F-BCA mice and their littermates. Scale bar: 200 μm. Data are presented as mean ± SEM. Statistical analyses were performed with two-tailed unpaired student t test. ***p < 0.001. Underlying data can be found in S1 Data. NS, not significant. F-BCA, Fsp1-Cre;Ctnnb1exon 3 fl/+; FSP1, fibroblast-specific protein-1; HE, hematoxylin–eosin; WAT, white adipose tissue. (TIF) [file pbio.2001493.s003.tif]

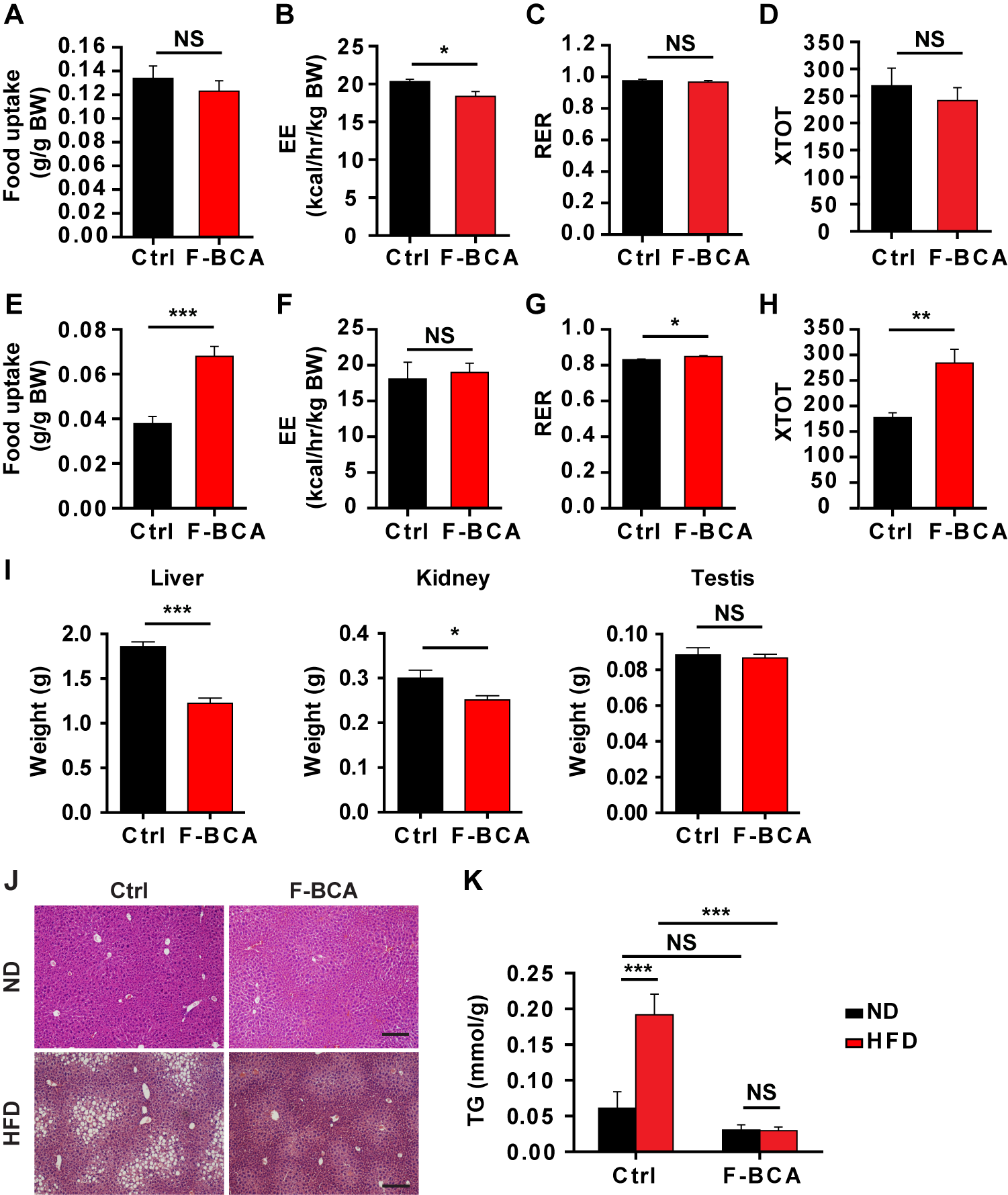

Supplement: S4 Fig — (A–H) Metabolic cage analyses were performed on F-BCA mice on ND (panel A–D) or on HFD (panel E–H). Food consumption (panel A and E), EE (panel B and F), RER (panel C and G), and XTOT (panel D and H) were recorded. n = 6 for each group. (I) Weight of liver, kidney, and testis of the F-BCA mice and their littermates at 4 months of age (liver: 13 Ctrl, 12 F-BCA; kidney: 6 Ctrl, 8 F-BCA; testis: 6 Ctrl, 6 F-BCA). (J) HE staining of liver sections of 4-month-old mice on ND or HFD. Scale bar: 200 μm. (K) Hepatic triglyceride levels in mice on ND or HFD. n = 5 for each group. Data are presented as mean ± SEM. Statistical analyses were performed with two-tailed unpaired student t test or one way ANOVA followed by Bonferroni's multiple comparison test. *p < 0.05; **p < 0.01; ***p < 0.001. Underlying data can be found in S1 Data. Ctrl, control; EE, energy expenditure; F-BCA, Fsp1-Cre;Ctnnb1exon 3 fl/+; FSP1, fibroblast-specific protein-1; HFD, high-fat diet; ND, normal-chow diet; NS, not significant; RER, respiratory exchange ratio; XTOT, physical activity. (TIF) [file pbio.2001493.s004.tif]

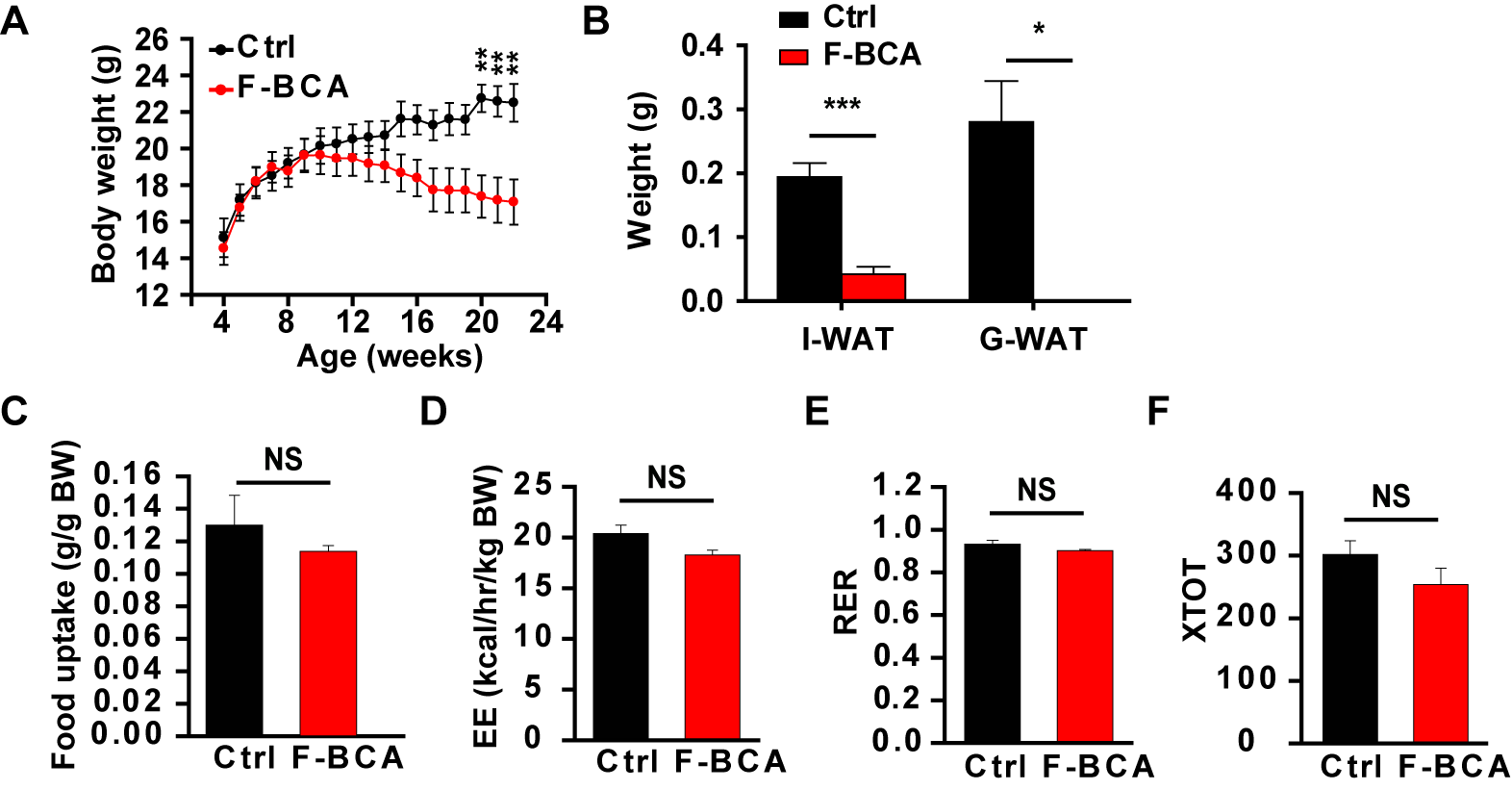

Supplement: S5 Fig — (A) Body weight of female control and F-BCA mice on ND. n = 8 for female control mice, and n = 8 for female F-BCA mice. (B) Weight of the adipose tissues of female control and F-BCA mice on ND at 4 months of age (I-WAT: 5 Ctrl, 9 F-BCA; G-WAT: 4 Ctrl, 7 F-BCA). (C–F) Metabolic cage analyses were performed on female control and F-BCA mice on ND. Food consumption (panel C), EE (panel D), RER (panel E), and XTOT (panel F) were recorded. n = 7 for female control mice, and n = 5 for female F-BCA mice. Data are presented as mean ± SEM. Statistical analyses were performed with two-tailed unpaired student t test or two-way ANOVA followed by Bonferroni's multiple comparison test (panel A). *p < 0.05; **p < 0.01. Underlying data can be found in S1 Data. Ctrl, control; EE, energy expenditure; F-BCA, Fsp1-Cre;Ctnnb1exon 3 fl/+; FSP1, fibroblast-specific protein-1; G-WAT, gonadal white adipose tissue; I-WAT, inguinal white adipose tissue; ND, normal-chow diet; NS, not significant; RER, respiratory exchange ratio; XTOT, physical activity. (TIF) [file pbio.2001493.s005.tif]

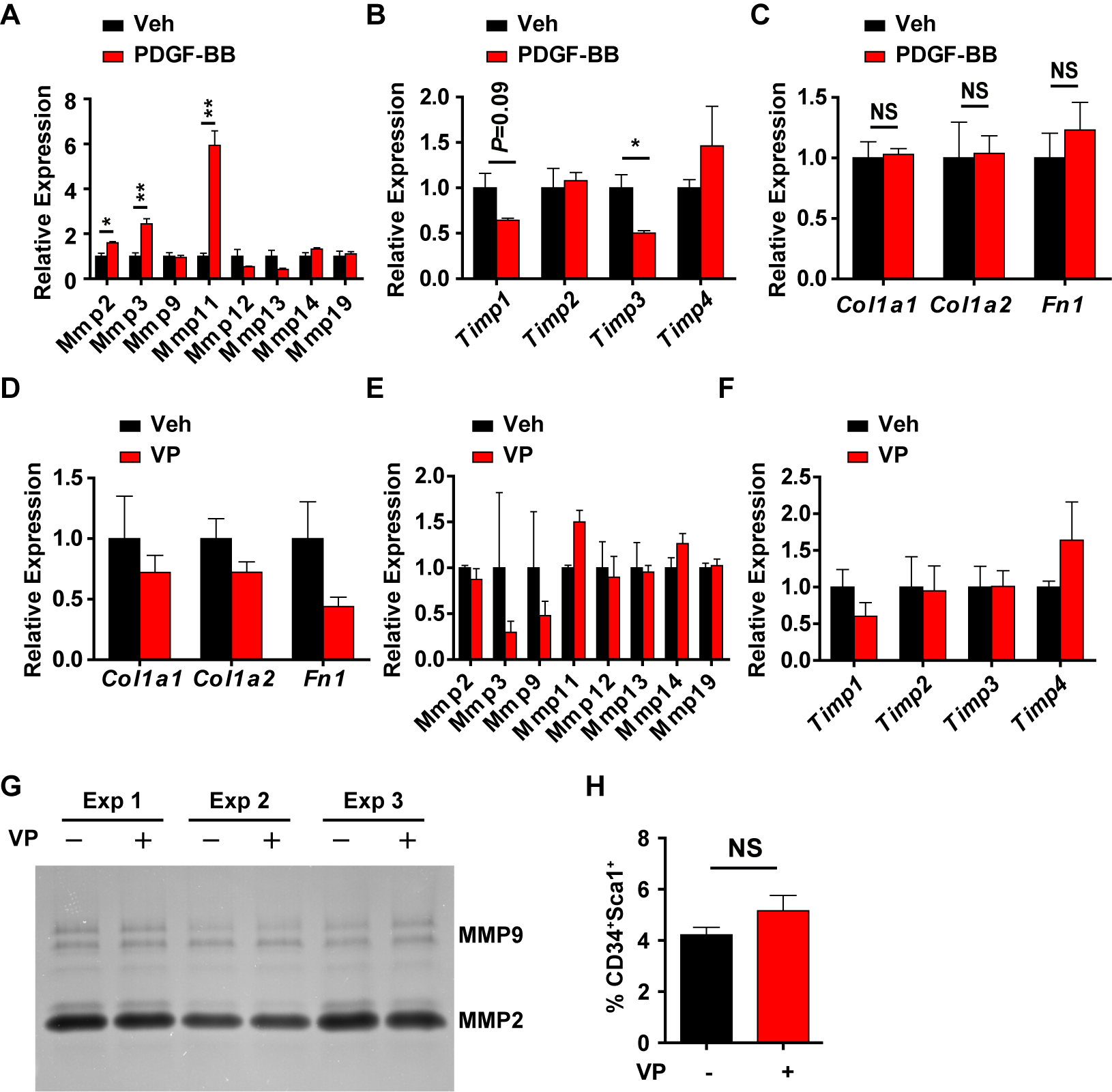

Supplement: S6 Fig — (A–F) RT-PCR analyses of expression of extracellular matrix proteins (panel C and D), MMPs (panel A and E) and TIMPs (panel B and F) in F-BCA SVF cells treated with or without 10 ng/mL PDGF-BB (panel A and C) or 0.5 μg/mL VP (panel D and F) (n = 3). (G) Gelatin zymography of conditioned medium of F-BCA SVF cells treated with or without 0.5 μg/mL VP. (H) SVF cells isolated from F-BCA I-WAT were treated with 0.5 μg/mL VP for 4 days before FACS analyses of CD34+Sca1+ populations (n = 3). Data are presented as mean ± SEM. Statistical analyses were performed with two-tailed paired student t test. *p < 0.05; **p < 0.01. Underlying data can be found in S1 Data. CD34, cluster of differentiation 34; FACS, fluorescence-activated cell sorting; F-BCA, Fsp1-Cre;Ctnnb1exon 3 fl/+; FSP1, fibroblast-specific protein-1; I-WAT, inguinal white adipose tissue; MMP, matrix metalloproteinase; NS, not significant; PDGF, platelet-derived growth factor; Sca1, stem cell antigen-1; SVF, stromal vascular fraction; TIMP, tissue inhibitor of metalloproteinase; VP, verteporfin. (TIF) [file pbio.2001493.s006.tif]

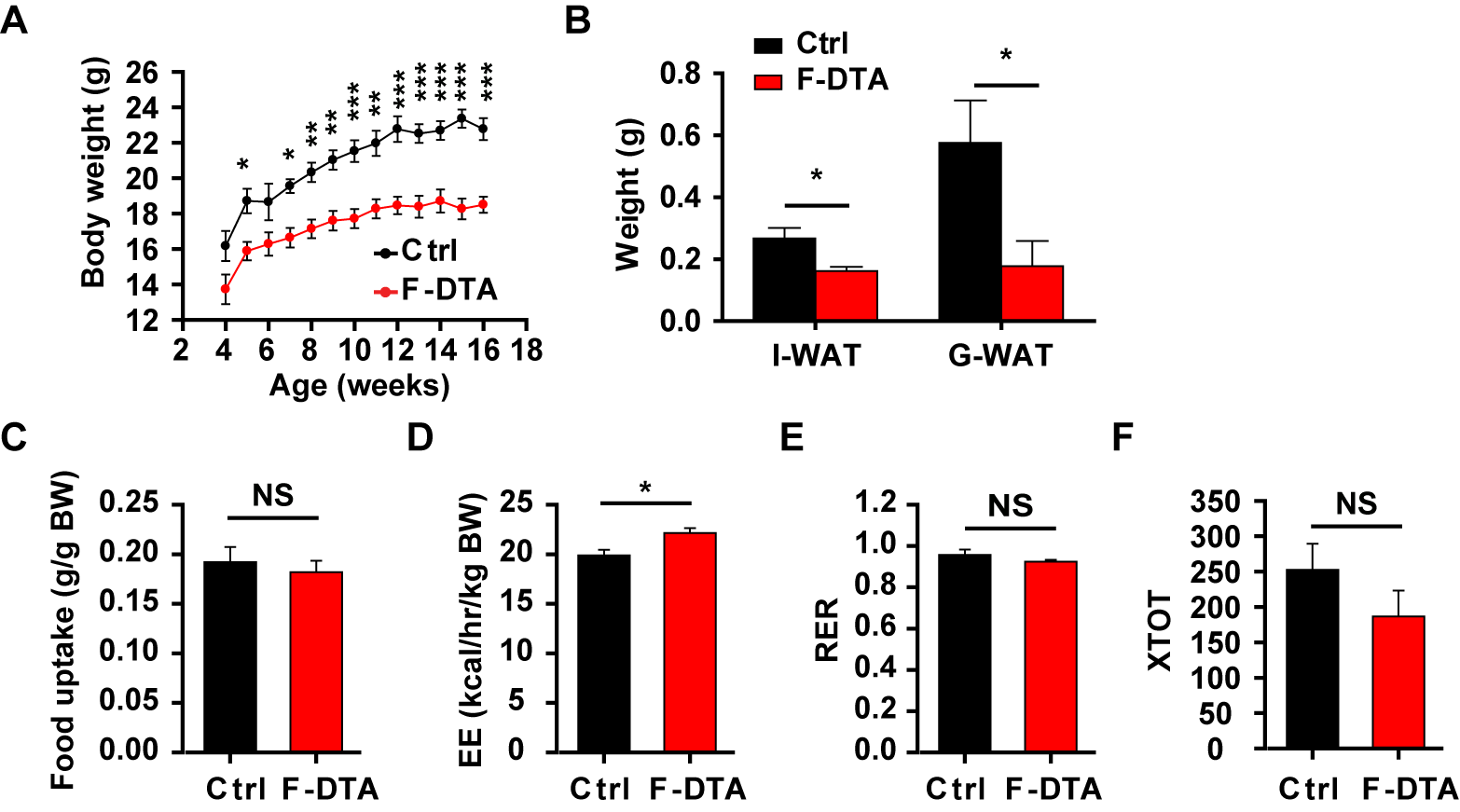

Supplement: S7 Fig — (A) Body weight of female control and F-DTA mice on ND. n = 7 for Ctrl mice and n = 7 for F-DTA mice. (B) Weight of the adipose tissues of female control and F-DTA mice on ND at 4 month age. n = 8 for female Ctrl mice, and n = 6 for female F-DTA mice. (C–F) Metabolic cage analyses were performed on control and F-DTA female mice on ND. Food consumption (panel C), EE (panel D), RER (panel E), and XTOT (panel F) were recorded. n = 6 for female Ctrl mice, except n = 7 for food consumption and n = 4 for female F-DTA mice. Data are presented as mean ± SEM. Statistical analyses were performed with two-tailed unpaired student t test (panel B–F) or two-way ANOVA followed by Bonferroni's multiple comparison test (panel A). *p < 0.05; **p < 0.01. Underlying data can be found in S1 Data. Ctrl, control; EE, energy expenditure; F-DTA, Fsp1-Cre;Rosa26-DTA; FSP1, fibroblast-specific protein-1; ND, normal-chow diet; NS, not significant; RER, respiratory exchange ratio; XTOT, physical activity. (TIF) [file pbio.2001493.s007.tif]

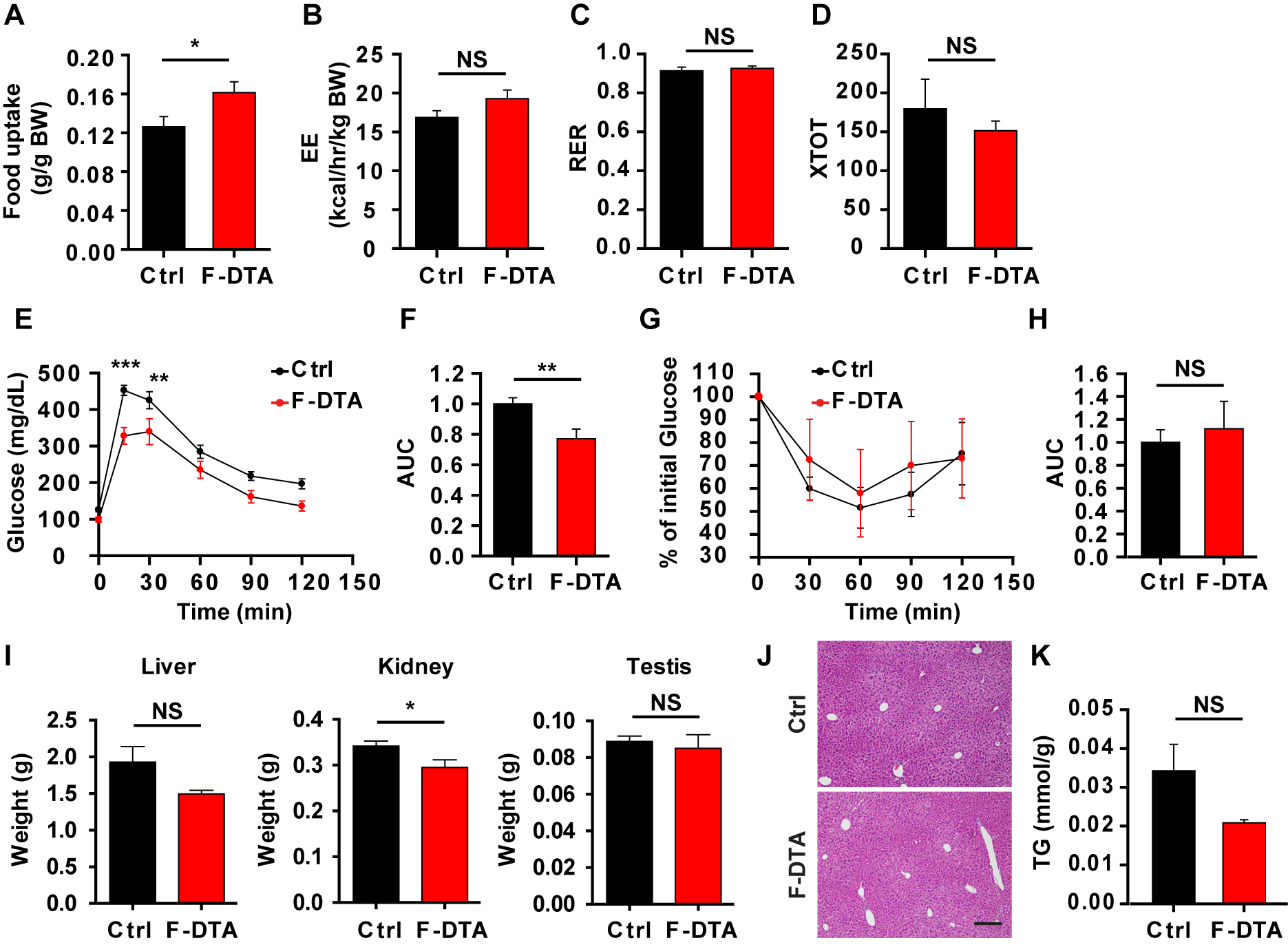

Supplement: S8 Fig — (A–D) Metabolic cage analyses were performed on male control and F-DTA mice. Food consumption (panel A), EE (panel B), RER (panel C), and XTOT (panel D) were recorded. n = 6 for each group, except n = 5 for food consumption. (E) GTT of male control and F-DTA mice at 4 months of age. n = 14 for male control mice; n = 10 for male F-DTA mice. (F) Quantification of the AUC of the GTT in panel E. (G) ITT of male control and F-DTA mice at 4 months of age. n = 10 for male control mice; n = 6 for male F-DTA mice. (H) Quantification of the AUC of the ITT in panel G. (I) Weight of liver, kidney, and testis of the 4-month-old male control and F-DTA mice (liver: 10 Ctrl, 11 F-DTA; kidney: 6 Ctrl, 6 F-DTA; testis: 8 Ctrl, 8 F-DTA). (J) HE staining of liver sections of 4-month-old male control and F-DTA mice. Scale bar: 200 μm. (K) Hepatic triglyceride levels in 4-month-old male control and F-DTA mice. n = 5 for each group. Data are presented as mean ± SEM. Statistical analyses were performed with two-tailed unpaired student t test or two-way ANOVA followed by Bonferroni's multiple comparison test (panel E and G). *p < 0.05; **p < 0.01; ***p < 0.001. Underlying data can be found in S1 Data. AUC, area under the curve; EE, energy expenditure; F-DTA, Fsp1-Cre;Rosa26-DTA; FSP1, fibroblast-specific protein-1; GTT, glucose tolerance test; HE, hematoxylin–eosin; ITT, insulin tolerance test; NS, not significant; RER, respiratory exchange ratio; XTOT, physical activity. (TIF) [file pbio.2001493.s008.tif]

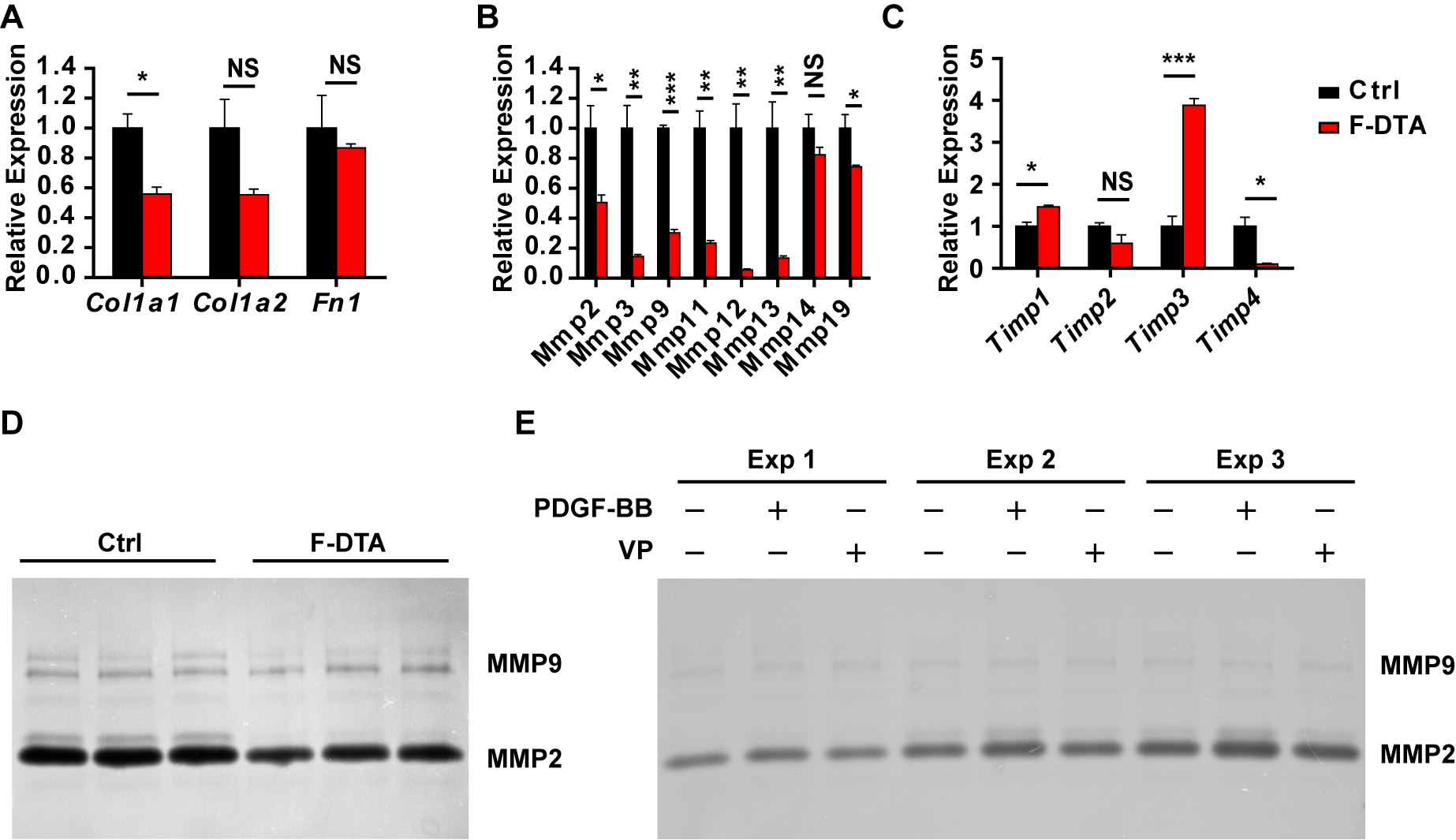

Supplement: S9 Fig — (A–C) RT-PCR analyses of expression of extracellular matrix proteins (panel A), MMPs (panel B), and TIMPs (panel C) in F-DTA and control SVF cells (n = 3). (D) Gelatin zymography of conditioned medium of F-DTA and control SVF cells. (E) Gelatin zymography of conditioned medium of F-DTA treated with or without 10 ng/mL PDGF-BB or 0.5 μg/mL VP. Data are presented as mean ± SEM. Statistical analyses were performed with two-tailed unpaired student t test. *p < 0.05; **p < 0.01; ***p < 0.001. Underlying data can be found in S1 Data. F-DTA, Fsp1-Cre;Rosa26-DTA; FSP1, fibroblast-specific protein-1; MMP, matrix metalloproteinase; NS, not significant; PDGF, platelet-derived growth factor; RT-PCR, reverse transcription PCR; SVF, stromal vascular fraction; TIMP, tissue inhibitor of metalloproteinase; YAP, Yes-associated protein. (TIF) [file pbio.2001493.s009.tif]
