# Supplementary material for: FSP1-positive fibroblasts are adipogenic niche and regulate adipose homeostasis
Source: PLoS Biol. 2018 Aug 6;16(8):e2001493. doi: 10.1371/journal.pbio.2001493 (PMC6078284; doi:10.1371/journal.pbio.2001493)
Supplement: S1 Table — qRT-PCR, quantitative reverse transcription PCR. (DOCX) [file pbio.2001493.s010.docx]

**Table S1. Primers used for qRT-PCR.**

| *Fabp2* | Forward | AAATGGGCATTAATGTGATGAA |
| --- | --- | --- |
|  | Reverse | ACACCGAGCTCAAACACAAC |
| *Pparg* | Forward | ACGTTCTGACAGGACTGTGT |
|  | Reverse | CTGTGTCAACCATGGTAATTTCAGT |
| *Cebpa* | Forward | TACCGAGTAGGGGGAGCAAA |
|  | Reverse | TCATTTTTCTCACGGGGCCA |
| *Fasn* | Forward | GTCCCGTCCAGTTCGCCTGC |
|  | Reverse | ACCTCCTCCATGGCTCTTCTCTGT |
| *Srebf1* | Forward | AAAGCTTGGCCTCCCAGCAGCC |
|  | Reverse | AGTGTGGCTGCAGTACAACTGGG |
| *Lipe* | Forward | CTCTTTACCGGTGGCCGATT |
|  | Reverse | ATGTTGGCCAGAGACGACAG |
| *Pnpla2* | Forward | TCACCATCCGCTTGTTGGAG |
|  | Reverse | GAAGGCAGATGGTCACCCAA |
| *Col1a1* | Forward | GGAAGAGCGGAGAGTACTGG |
|  | Reverse | GGCTGAGTAGGGAACACACA |
| *Col1a2* | Forward | TGGTCTTACTGGGAACTTTGCTGC |
|  | Reverse | ACCCTGTGGTCCAACGACTCCTCTC |
| *Fn1* | Forward | CCAGAAACAGATGCAACGAT |
|  | Reverse | GCAGACACACTGAAGCAGGT |
| *Mmp2* | Forward | CAACACTGGGACCTGTCACT |
|  | Reverse | CCAAATAAACCGGTCCTTGA |
| *Mmp3* | Forward | ATGGGCCTGGAACAGTCTTG |
|  | Reverse | GGTTGGTACCAGTGACATCCTC |
| *Mmp9* | Forward | TTTGAGTCCGGCAGACAAT |
|  | Reverse | CTTCCAGTACCAACCGTCCT |
| *Mmp11* | Forward | TGCATTCAGGGGTGATTCAGA |
|  | Reverse | GCCTTTGAGGTTCCGTGTCT |
| *Mmp12* | Forward | TTGTGGATAAACACTACTGGAGGT |
|  | Reverse | AAATCAGCTTGGGGTAAGCA |
| *Mmp13* | Forward | GGAGCCCTGATGTTTCCCAT |
|  | Reverse | GTCTTCATCGCCTGGACCATA |
| *Mmp14* | Forward | GCCCTCTGTCCCAGATAAGC |
|  | Reverse | ACCATCGCTCCTTGAAGACA |
| *Mmp19* | Forward | GCAAAGACCTGGAGGATTACCT |
|  | Reverse | GCCTCTGTGATATCTTCCAGCC |
| *Timp1* | Forward | GCAAAGAGCTTTCTCAAAGACC |
|  | Reverse | AGGGATAGATAAACAGGGAAACACT |
| *Timp2* | Forward | AGGTACCAGATGGGCTGTGA |
|  | Reverse | GTCCATCCAGAGGCACTCAT |
| *Timp3* | Forward | GGCTTCAGTAAGATGCCCCA |
|  | Reverse | CTTCATACACGCGCCCTGTC |
| *Timp4* | Forward | GGCTGCCAAATCACCACTTG |
|  | Reverse | TGGGCCTGGTACCCATAGAG |
| *Yap1* | Forward | TTTCGGCAGGAATTAGCTCT |
|  | Reverse | GAGAAGACACTGCATTCGGA |
| *Ctgf* | Forward | GAGTGTGCACTGCCAAAGAT |
|  | Reverse | GGCAAGTGCATTGGTATTTG |
| *Ankrd1* | Forward | CCAGAGACAAGCTTCTCAGCA |
|  | Reverse | TGGCATTGAGATCAGCCTCG |
| *Pdgfa* | Forward | AGGAGGAGACAGATGTGAGGT |
|  | Reverse | TTCAGGAATGTCACACGCCA |
| *Pdgfb* | Forward | GGAGTCGGCATGAATCGCT |
|  | Reverse | GAATGGGATCCCCCTCGG |
| *Cd34* | Forward | TACAGGAGAAAGGCTGGAGCTG |
|  | Reverse | TGAGGAGAGCACAAAGGAAGT |
| *Ly6e* | Forward | GGTGCATTCAGCCTTTGGTG |
|  | Reverse | CCAAAGAAGCCTACTCCGGG |
